# A Systematic Review on the Potential Acceleration of Neurocognitive Aging in Older Cancer Survivors

**DOI:** 10.3390/cancers15041215

**Published:** 2023-02-14

**Authors:** Charlotte Kerstens, Hans P. M. W. Wildiers, Gwen Schroyen, Mercedes Almela, Ruth E. Mark, Maarten Lambrecht, Sabine Deprez, Charlotte Sleurs

**Affiliations:** 1 Department of Oncology, University Hospital Ghent, 9000 Ghent, Belgium; 2 Leuven Cancer Institute, KU Leuven, 3000 Leuven, Belgium; 3 Department of General Medical Oncology and Multidisciplinary Breast Centre, University Hospitals Leuven, 3000 Leuven, Belgium; 4 Laboratory of Experimental Oncology (LEO), Department of Oncology, KU Leuven, 3000 Leuven, Belgium; 5 Leuven Brain Institute, KU Leuven, 3000 Leuven, Belgium; 6 Department of Imaging and Pathology, Translational MRI, KU Leuven, 3000 Leuven, Belgium; 7 Department of Cognitive Neuropsychology, Tilburg University, 5030AB Tilburg, The Netherlands; 8 Laboratory of Experimental Radiotherapy, Department of Oncology, KU Leuven, 3000 Leuven, Belgium; 9 Department of Radiotherapy, University Hospitals Leuven, 3000 Leuven, Belgium

**Keywords:** neurodegeneration, cognition, aging, older, cancer survivors

## Abstract

**Simple Summary:**

As survival rates for cancer increase and most patients exceed the age of 65 years, more emphasis has gone to possible cognitive sequelae, which could be explained by accelerated brain aging. We conducted a systematic literature review to summarize the existing risks of cognitive decline, imaging-based indication of neurotoxicity, as well as developing a neurodegenerative disease in older cancer survivors. Evidence was found for functional and structural brain changes. Cognitive decline was mainly found in memory functioning. Individual risk factors included cancer types (brain, hormone-related cancers), treatment (anti-hormonal therapy, chemotherapy, cranial radiotherapy), genetic predisposition (APOE, COMT, BDNF), increasing age, comorbidities (frailty, baseline cognitive reserve, functional decline), and psychological (distress, depression, anxiety, post-traumatic stress disorder, sleeping problems, fatigue) and social factors (loneliness, caregiver support, socioeconomic status). Further research is needed to provide a more detailed and profound picture of accelerated neurocognitive aging in specific older subpopulations and targeted interventions.

**Abstract:**

As survival rates increase, more emphasis has gone to possible cognitive sequelae in older cancer patients, which could be explained by accelerated brain aging. In this review, we provide a complete overview of studies investigating neuroimaging, neurocognitive, and neurodegenerative disorders in older cancer survivors (>65 years), based on three databases (Pubmed, Web of Science and Medline). Ninety-six studies were included. Evidence was found for functional and structural brain changes (frontal regions, basal ganglia, gray and white matter), compared to healthy controls. Cognitive decline was mainly found in memory functioning. Anti-hormonal treatments were repeatedly associated with cognitive decline (tamoxifen) and sometimes with an increased risk of Alzheimer’s disease (androgen deprivation therapy). Chemotherapy was inconsistently associated with later development of cognitive changes or dementia. Radiotherapy was not associated with cognition in patients with non-central nervous system cancer but can play a role in patients with central nervous system cancer, while neurosurgery seemed to improve their cognition in the short-term. Individual risk factors included cancer subtypes (e.g., brain cancer, hormone-related cancers), treatment (e.g., anti-hormonal therapy, chemotherapy, cranial radiation), genetic predisposition (e.g., APOE, COMT, BDNF), age, comorbidities (e.g., frailty, cognitive reserve), and psychological (e.g., depression, (post-traumatic) distress, sleep, fatigue) and social factors (e.g., loneliness, limited caregiver support, low SES). More research on accelerated aging is required to guide intervention studies.

## 1. Introduction

In 2020, the worldwide incidence of cancer was 19.3 million [1]. Thanks to improvement in treatments, as well as earlier detection, survival rates have increased, resulting in more long-term and older survivors of cancer [2,3,4]. Hence, quality of life in survivorship of these patients has become an important topic in cancer research. While the older population of cancer survivors is the largest, most neurocognitive studies do not focus on this specific population. It is important to understand the long-term effects of cancer and its treatment on the neurocognitive aging process of this older population of survivors [2]. One such long-term effect entails neurocognitive decline, which is an important factor contributing to quality of life (QoL) [5]. Cognitive deficits are mostly summarized under the term “cancer-related cognitive impairment (CRCI)”. CRCI was described earlier by Janelsins and colleagues (2014) as immediate or delayed cognitive difficulties after cancer and its treatment, including perceived and objective decline in memory, attention, concentration, and executive function [6].

The involved neurotoxic mechanisms can be treatment-specific (e.g., radiotherapy, chemotherapy, and immunotherapy), but overlap between these mechanisms exists. For instance, the possible mechanisms of chemotherapy-induced cognitive changes include decreased integrity of the blood–brain barrier, neuronal apoptosis and reduced neurogenesis, DNA damage, inflammation and cytokine deregulation, reduced estrogen and testosterone levels, cardiotoxic effects, neuroendocrine changes, and genetic predispositions [7]. More recently, Makale and colleagues (2017) reviewed possible central neurotoxic mechanisms of cranial irradiation. These similarly cover changes of neuronal apoptosis and reduced neurogenesis, inflammation and damage to neuronal dendrite structures, and prefrontal cortex damage (white matter, vessels, and neurons) [8]. Joly et al. (2020) studied potential central neurotoxic mechanisms of immunotherapy. Higher pro-inflammatory cytokines and growth factors, cytokine dysregulation, increase in T-cell receptor diversity, and white blood cell count could all have an adverse effect on immune-related events affecting all organs of the body. All these processes could indirectly cause neural degeneration as well. However, evidence regarding neuropsychological outcomes post-immunotherapy remains scarce [9,10].

In most people, the cognitive deficits and/or complaints tend to resolve within the first few months after treatment, but in about a third of survivors, the cognitive deficits and/or complaints can persist longer [11]. Confounding factors such as sociodemographic factors (e.g., age, cognitive reserve, socioeconomic status, education), genetics, physical conditions (e.g., comorbidities, frailty, postmenopausal status), and psychological factors (e.g., fatigue, emotional distress, allostatic load and lifestyle) can further explain daily life functioning [12,13,14]. The multifactorial nature causes some people to be more at risk for cognitive impairment than others and complicates the identification of responsible components for changes in cognition, which can even be more elevated in older patients [12,15].

Aging occurs throughout an individual’s lifespan. Normal biological aging involves the accumulation of damage on the molecular and cellular level over time, resulting in a deterioration of physical and mental capacities and an increased vulnerability to disease [15]. The cellular mechanisms involved in this process include DNA damage and mutations, epigenetic aging, stem cell damage (oxidative stress), cellular senescence (telomere shortening), and inflammation. Each of these can contribute to neurocognitive aging and cognitive decline [16]. Cancer, of which dysregulated cell growth is one of the hallmarks, shares some of the same mechanisms as aging. This could clarify the bidirectional relationship between cancer and aging [16].

The goal of this review is to better understand the potential central neurotoxic effects of cancer and its treatment on the neurocognitive aging process in older cancer survivors. To address this aim in a comprehensive way, we summarized the existing literature on neuroimaging, neuropsychological functioning, and neurodegenerative disorders in this population.

## 2. Method

### 2.1. Search Strategy

A literature search was conducted in PubMed, Web of Science, and MEDLINE databases. See Supplementary S1 for an overview of the searches in the search engines. The search included articles exclusively in English, dated between 1 January 2000 and 12 June 2021. The three main key search terms selected were related to ‘Neoplasm’ *and* [‘Neurocognition’ *or* ‘Neurodegeneration’] *and* ‘Elderly’. Synonyms were searched based on the database-trees and added for each key term. MeSH-terms were utilized where available. See Supplementary S2 for the detailed search string. This systematic review is registered at: https://doi.org/10.17605/OSF.IO/TBDFP (accessed on 17 January 2023).

### 2.2. Eligibility Criteria

Studies considered for review included (1) research on cancer survivorship starting 6 months after the last treatment or at least 1 year post-diagnosis; (2) a mean age of at least 65 years old at the moment of testing; (3) central neurotoxic changes (i.e., inflammation, hypo- or hyper-brain activation, structural and functional brain changes), neurocognitive symptoms (deficits or complaints) and neurodegenerative disorders (e.g., dementia diagnoses); (4) original studies; (5) human studies; (6) non-palliative treatment; and (7) studies without neuropsychological interventions. Excluded studies were (1) studies with data acquisition during treatment, or within 6 months after treatment; (2) younger average age than 65 years old; (3) no neurocognitive or neurological outcomes were measured; (4) non-original-research articles (case reports, expert opinions, conference summaries), reviews, meta-analysis, protocols, case studies (i.e., ≤5 patients); (5) in vitro or animal studies; (6) palliative population; and (7) post-treatment neuropsychological intervention studies.

### 2.3. Data Extraction

Duplicate articles were removed through EndNote and uploaded to Rayyan as a screening measure. Studies were then categorized according to the measurements used to determine functioning (i.e., imaging studies, neuropsychological tests, or questionnaires/interviews). Some studies used a combination of measurements (imaging, cognitive testing, interviews/questionnaires) in their analysis. These studies were categorized based on the focus of the study (Appendix A Table A1 imaging studies, Table A2 cognitive studies, Table A3 questionnaire studies). Specific neurodegenerative diseases (e.g., Alzheimer’s disease, Parkinson’s disease, other dementia types or cerebrovascular conditions) following cancer or its treatment were separately described (Appendix B Table A4).

Information of author, publication year, study-design, cancer subtype, treatment type, comparison group, participants, mean age at diagnosis, mean age at baseline, measurements used, and the main findings relevant to the current protocol were extracted per study (Table A1, Table A2 and Table A3).

## 3. Results

### 3.1. Study Characteristics

**Figure 1:**
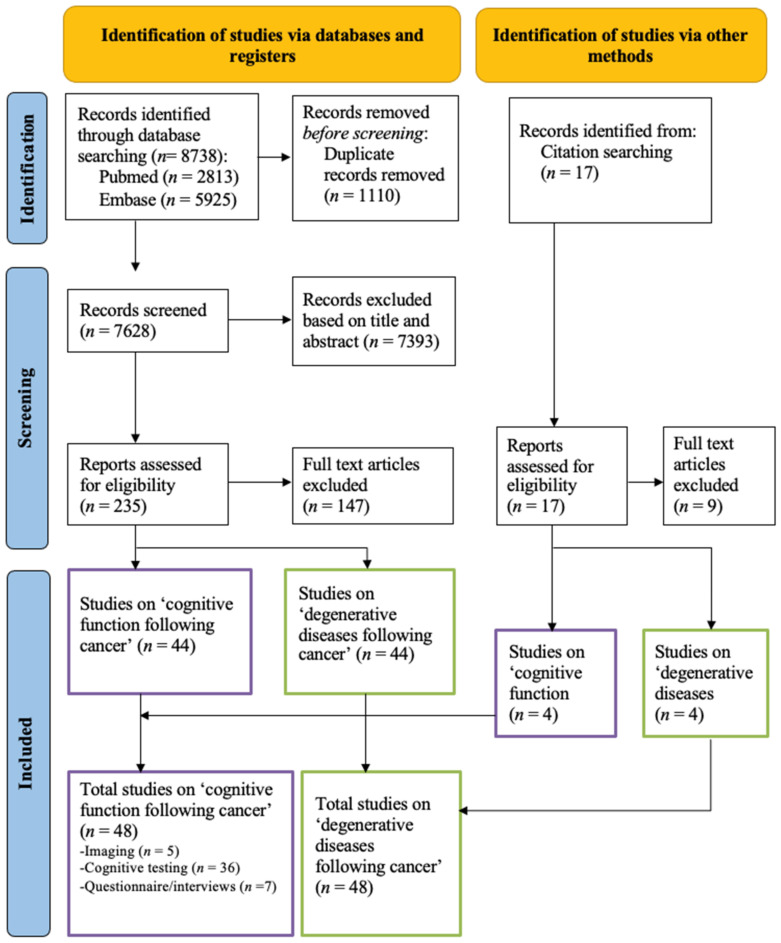
PRISMA 2020 flow diagram.

The search identified a total of 8738 citations, of which 2813 were in the search engine Pubmed, and 5925 in Web of Science (Figure 1). The duplicate articles were eliminated using EndNote, resulting in 7628 remaining articles. These studies were then uploaded to Rayyan and evaluated based on their title and abstract, and 7393 articles were excluded. Of the remaining articles, 235 were evaluated in detail. Four additional articles were identified by manually searching for studies that have cited these papers. This resulted in a final selection of 48 publications relating to the research question, which were divided into the different outcome categories (i.e., imaging studies, cognitive tests, questionnaires/interviews). Five studies (10%) primarily described imaging findings. Thirty-six studies (75%) assessed the neurocognitive impact of cancer and its treatment based on cognitive testing and seven (15%) based on questionnaires/interviews. Fourteen studies used a combination of measurements (imaging, cognitive testing, interviews/questionnaires) in their analysis. Forty-eight studies explicitly reported on neurodegenerative diseases after cancer treatment, which are separately described (Figure 1).

### 3.2. Imaging Studies

Normal brain aging includes decreases in total brain volume, gray and white matter connectivity, and hippocampus volume changes [17]. Structural changes in the brain were found in older survivors of cancer compared to age- and education matched controls. These included gray matter volume loss in areas such as the basal ganglia and right superior frontal gyrus [18,19,20] and white matter changes in the corpus callosum, exceeding the normal aging process. These brain changes correlated with overall cognitive impairment as well as specific cognitive functions such as language processing, verbal fluency, processing speed, executive functions, visuospatial abilities, visual and verbal memories, and word recall [20].

In addition, functional changes in brain metabolism were also found in survivors after chemotherapy, chemoradiation, or tamoxifen treatment. These included hypometabolism in orbital frontal regions and hypermetabolism in the left postcentral gyrus, which correlated with worse executive functioning, working memory, and divided attention. These changes could reflect potential dysfunction in frontal-subcortical brain regions [21]. Hypoactivation of frontal areas is also seen in normal aging. Treatment can thus accelerate or mimic the effects of normal cognitive aging in survivors [22]. Interestingly, lower concentrations of myo-inositol were found in the brain after tamoxifen or estrogen treatments, while normal aging is associated with increased concentrations of myo-inositol, suggesting that brain aging might be favorably modulated by specific anti-hormone therapies [23]. Specifically, the most affected regions in cancer survivors were the frontal regions and changes in the basal ganglia consistent with regions affected by normal neurocognitive aging [18,20,21,23,24].

### 3.3. Neuropsychological Testing

A broad spectrum of tests (*n* = 36) measuring different cognitive domains were used in the studies (including attention, memory, processing speed, executive functioning, learning, language, visuospatial abilities, reaction time, psychomotor function, intelligence, and non-verbal function). Treatment-specific results were most often found.

Regarding CNS tumors, most often neurosurgery showed improvements in cognitive function [25,26]. One study showed no cognitive change after stereotactic radiotherapy for brain metastases [27], while another revealed lower or impaired cognitive scores more than 9 months after focal irradiation for glioblastoma [28].

In non-CNS tumor patients, local therapy (surgery or radiotherapy) did not have a substantial impact on cognition [29,30,31,32,33,34]. Only for sinonasal and gynecological cancers, impaired cognitive functioning was found after radiotherapy and/or surgery [19,35]. This can be explained as irradiation for sinonasal cancer, which is located close to the brain, which could indirectly affect the brain [19]. For the study on gynecologic cancers, local therapy, directly affected ovarian function, resulting in decline in estrogen levels, thus similarly affecting cognition as was seen by the studies on anti-hormone therapy [35].

Anti-hormonal therapy, specifically tamoxifen, resulted in worse learning (information processing), verbal memory, and executive functioning in cancer survivors compared to healthy controls [36,37,38,39]. ADT in prostate cancer resulted in worse cognitive performance, compared to healthy controls, specifically in executive function attention, memory, and information processing [30,40,41,42,43], although not consistently replicated [44,45,46,47,48]. These deficits related to anti-hormonal therapy are strongest and most often found during and shortly after treatment [38,40]. A potential cognitive benefit was found of exogenous levothyroxine in thyroid cancer on the cognitive function of patients who lack endogenous thyroid hormone [49].

Chemotherapy negatively impacted cognitive processing speed, visual and verbal memory, spatial function, and attention in most studies [24,50,51,52,53], although not consistently replicated [31,33,54]. One study found better recall in survivors that had received chemotherapy, but this was due to an age and treatment interaction as younger people were more often in better conditions and received chemo [32].

Inconsistent results were found when comparing studies that did not distinguish between specific treatments and/or cancer types. Some found similar trajectories of cognitive functioning compared to healthy controls [31,55,56], others found that cancer survivors in general have more cognitive impairment [57,58]. One study even showed spurious results of better memory and slower memory decline in older cancer survivors compared to healthy controls [59].

Generally, older age or aging-related phenotypes such as frailty were associated with worse cognition scores and impairment [28,31,33,36,37,51,53,56]. Depression/anxiety and fatigue were also found to predict worse cognition [31,56,60].

### 3.4. Interviews/Questionnaires

Divergent results were found in the studies on subjective cognitive complaints. There were studies that found no association between previous cancer diagnosis and self-reported cognitive complaints, maintaining good long-term self-reported cognitive complaints [61,62,63]. Two studies showed the opposite, that long-term survivors most often presented a higher rate of cognitive complaints [42,64]. Memory domains were most likely perceived as affected, specifically the ability to learn new information [38,42,58,65]. Loss of memory was more often reported in female breast cancer survivors or gynecologic cancers [35,66], and in patients with pre-existing cognitive or memory complaints [63,65]. One study investigated anti-hormonal therapy and found that tamoxifen users (but not exemestane users) reported increased attention/concentration complaints [60]. Chemotherapy studies more frequently reported loss of memory [65,66] or worse perceived concentration, or general cognitive abilities [36,63], although not always replicated [61].

### 3.5. Neurodegenerative Diseases following Cancer

Treatment- and cancer-specific results were most often found. Most studies found a decreased risk for AD or a delay in onset (but not progression) of PD in patients with skin cancer [18,67,68,69]. Two studies found that skin cancer increased the risk of AD [70] or PD [71]. Divergent results were found for smoking-related cancers (i.e., lung, oral, larynx, pharynx, esophagus, stomach, pancreas, bladder, kidney, and cervical cancer). While some studies found a decreased risk of AD, PD, or stroke in these cancers [70,71,72,73,74], others found the opposite [75,76,77]. Hormone-related tumors (i.e., breast, uterus, and prostate cancer) were associated with decreased risk of developing AD or PD in some studies [76,78,79] while other studies found an increased risk [70] or no association [80,81].

In addition, differential effects for cancer treatments were found. The majority of studies found that the use of ADT resulted in increased risk of developing AD compared to no ADT treatment [82,83,84,85,86,87,88,89,90], with increasing risk in case of longer use [88], although not consistently replicated for AD or PD [91,92,93,94] or for cerebral infarction [95]. Regarding anti-hormonal therapy for breast cancer, one study found that aromatase inhibitors resulted in less risk of dementia than tamoxifen treatment [96] while another study found no difference between both treatments in the risk for dementia [97]. One study used a comparison group of no anti-hormonal treatment and found that tamoxifen and aromatase inhibitors were associated with decreased risk of AD and dementia [98]. Other treatments such as Bacillus Calmette–Guerin also showed reduced risk of AD and PD [73].

In comparison to radiotherapy in head and neck cancers, surgery had a comparable risk of consequent cerebrovascular events in one study [99], while in another, higher rates of cerebrovascular events were found in patients receiving radiotherapy compared to surgery alone [100]. One study showed that the use of some statins after radiotherapy could reduce this risk [101].

Some studies showed chemotherapy to be related to drug-induced dementia [102,103], while the risk of other types of dementia such as AD and vascular dementia were lower in patients that received chemotherapy [70,102]. Other studies found no associations [104,105,106,107].

When comparing studies that did not distinguish between specific treatments and/or cancer types, the majority of studies found that older cancer survivors have a lower risk of developing Alzheimer’s disease (AD) compared to healthy controls [18,72,74,78,79,80,108,109,110]. Some studies found no relevant association between cancer and risk of dementia or transient global amnesia [76,111,112,113]. Comorbid factors such as socio-economic status and depression increased the risk of dementia [106,107].

## 4. Discussion

Given the rising incidence of cancer with age, research on the neurocognitive and neurodegenerative impact of cancer and its treatment in later life is important. Only a relatively small number of studies have focused on cancer survivors with a higher biological age. Overall, evidence was provided for functional and structural changes in the brain, specifically gray and white matter changes in the frontal regions and basal ganglia, consistent with changes in cognition, specifically working memory, executive functioning, and information processing. Anti-hormonal treatments were repeatedly associated with worse cognition (including tamoxifen) and sometimes with an increased risk of developing Alzheimer’s disease (regarding ADT). Similarly, chemotherapy inconsistently resulted in cognitive changes or drug-induced dementia. Local surgery or radiotherapy was not associated with cognition in patients with non-CNS cancer. By contrast, local radiation to the head (cranial radiotherapy) did seem to play a cognitive role in patients with CNS cancer. For these patients, neurosurgery seemed to improve their cognition in the short-term.

Across studies, memory was frequently perceived as affected. When looking at the studies on neurodegenerative conditions in cancer survivors’, divergent results were found for skin cancer and smoking- and hormone-related cancers, some increasing the risk of dementia and others showing a decrease in risk. When focusing on studies that did not distinguish between specific treatments or cancer types, most of these studies found that older cancer survivors have a lower risk of developing Alzheimer’s disease (AD) compared to healthy controls.

### 4.1. Individual Risk Factors

As results are diverse, possible individual risk factors can be important to consider. These can include cancer types, treatment, genetic predisposition, age, comorbidities, and psychological and social factors [57,114]. As frailty increases with aging, due to physical, psychological–emotional, and cognitive functional deterioration, patients can cognitively deteriorate. This could even be accelerated, given that cancer patients often suffer from physiological and emotional sequelae related to their diagnosis and treatment.

A cancer diagnosis and the personal context (e.g., fatigue, sleep problems, hormonal changes, and tumor-related factors) can have indirect effects on cognition, which could be even more pronounced in older compared to younger people [3,115]. Relatedly, genetic predisposition can influence the relationship between cancer and cognition or neurodegeneration, such as genes associated with age-related cognitive decline [22,116]. These include genes encoding apolipoprotein E (APOE), catechol-O-methyltransferase (COMT), and brain-derived neurotrophic factor (BDNF) [114].

In addition, age-related comorbidities and frailty are an important consideration as well, as chronological age alone appears to be a poor predictor of the effects of treatment [117]. The combination of chronological age or age-related phenotypes such as frailty or cognitive reserve could better predict neurodegeneration in cancer survivors [36,115]. Increasing age also leads to an accumulation of multimorbidity, functional decline, and cognitive dysfunction that may degenerate into dementia symptoms [115,118]. Thus, survivors who are older and have less reserve pre-diagnosis could be more susceptible to reaching the threshold of cognitive deficits [64].

Psychological and social factors can also be a risk factor in cancer-related cognitive impairment or neurodegeneration [114]. The prevalence of mood disorders such as depression or anxiety is known to be high in adults aged 65 and above compared to younger adults [3,114]. Older people more often have decreased social activities which provides additional emotional and practical challenges such as loneliness, needs of caregiver support, transportation, and home care [119]. There is a relationship between social isolation and increased cancer mortality as well as poorer treatment tolerance [119].

Each of these risk factors (cancer, treatment, genetic predisposition, age, comorbidities, psychological and social factors) can lead to physiological toxic effects and can affect the aging brain [114]. However, not all older cancer patients develop cognitive effects, and the risk can depend on individual resilience factors [22]. Interestingly, brain changes can also be found in older cancer patients without decline in neuropsychological functioning [120]. In some cancer patients overactivation in the brain was found, which could suggest compensatory mechanisms.

### 4.2. Physiological Features of Aging

Cancer and aging are linked biological processes, and the diagnosis of cancer and its treatment can accelerate the aging process [121]. An overlapping pathway involved in aging, cancer, and treatment are inflammatory responses as they can trigger neurotoxic cytokines [22].

First, hormonal levels decrease with age and can more profoundly decrease when anti-hormonal therapies are prescribed as cancer treatment [22]. Hence, different effects have been found, dependent on the type of anti-hormonal treatment. Second, treatment such as chemotherapy can disrupt cellular processes and cell division resulting in increased inflammatory responses [114]. DNA damage and diminished DNA repair are markers of senescence and are found in age-related diseases such as PD, AS, and mild cognitive impairment [22]. Some chemotherapies have been shown to cross the blood–brain barrier and strengthen central neurotoxicity [22]. Telomere length is a marker of cellular age, stress, AD, cancer risk, and mortality. Certain cancer treatments influence telomere length, resulting in a common pathway between aging and cancer-related cognitive decline [22]. Senescent cells are also a biomarker of the frailty phenotypes that could increase the risk of cognitive decline [22].

These pathways can also result in overlapping brain changes affected by cancer treatment, neurocognitive aging, and neurodegeneration. While normal aging has a curvilinear process with most decline in older age, the slope can change due to individual risk factors [122]. Thus, even if cancer treatment has the same impact on the brain independent of age, the cognitive performance may change depending on the age of the individual and the slope of cognitive aging [122].

### 4.3. Gaps in Research and Future Directions

This review demonstrated that cancer diagnosis and treatment could have an adverse effect on cognition or neurodegeneration and inter-study differences were found. However, some limitations need to be mentioned. First, a common limitation in many studies was the relatively small sample size, raising questions on representativeness of the sample group in the general population. Second, studies often included selected sub-populations (e.g., excluding patients with too much comorbidity), which could result in a selection bias. For instance, the number of studies on patients with CNS tumors was limited. Most studies looked at the treatment of anti-hormone therapy or chemotherapy while the results on immune therapy and local therapy were limited. Third, different validated measures of cognitive function were used in different studies making it difficult to make a comparison. Given the wide scope of existing findings that we aimed to summarize, and the current lack of such comprehensive overview, we included both cognitive and dementia research to integrate different perspectives addressing potential accelerated neurocognitive aging. In this study, we conducted a systematic review covering different aspects. This was selected given that the existing data to date are too diverse and limited to perform a meta-analysis. More specifically, more than thirty different neuropsychological test materials were used to assess cognitive functioning, covering attention, memory, processing speed, executive functioning, learning, language, visuospatial abilities, reaction time, psychomotor function, intelligence, and non-verbal function. Moreover, different cancer populations were included, both non-CNS and CNS cancer types. The majority of studies covered either neuropsychological test assessments, or epidemiological studies on neurodegenerative diseases. Studies focusing on neuroimaging or questionnaire data only in the elderly population were rather limited.

A combination of imaging, cognitive testing, and subjective cognitive complaints gives the most information on the effects of cancer and treatment on cognition and neurodegeneration as some results may be very subtle. Fourth, not all studies described the different cancer treatments (or its timeline) in detail, which complicates the interpretation of treatment-specific effects. Finally, many studies did not use a control group without cancer (either healthy controls or cancer survivors who did not receive a specific treatment), making it difficult to compare to the general healthy population, thus concluding whether cancer and/or its treatment accelerated the normal aging process.

Future studies on the neurocognitive and neurodegenerative impact of cancer treatment should include sufficient numbers of older cancer survivors in order to capture variability in reserve and frailty and to highlight the effects of different treatments, biological processes and other chronic comorbidities.

Evaluating someone’s individual risk for developing short- or long-term cognitive deficits or neurodegeneration in later life is important in creating a treatment plan. This can be done through a multidimensional assessment, having a predictive value in identifying a subgroup of cancer patients and older survivors that are at higher risk for cognitive decline, thus needing closer monitoring and intervention. More imaging studies will be critical in identifying brain structural links between cancer and neurocognitive aging. Research on treatments that have less toxic impact and provide more quality of life are essential as well. For those patients that do experience cognitive decline and neurodegeneration, rehabilitation programs and interventions should be created to support these cognitive losses. Through the understanding of specific risk factors for cognitive deficits in older cancer survivors, and by understanding the link between cancer treatment and the neurocognitive aging process, tools could be developed to identify patients more at risk for accelerated neurocognitive aging, neurodegeneration, or cognitive dysfunctions.

## 5. Conclusions

In this review, we provided a comprehensive overview of evidence related to potential accelerated brain aging, including neuroimaging, and neurocognitive and neurodegenerative disorders studies in older cancer survivors (>65 years). Evidence was found for functional and structural brain changes in multiple areas (frontal regions, basal ganglia, gray and white matter). Cognitive decline was mainly found in memory. Anti-hormonal treatments were repeatedly associated with cognitive decline (tamoxifen) and sometimes also with Alzheimer’s disease (androgen deprivation therapy). Chemotherapy was inconsistently associated with later development of cognitive changes or dementia. Radiotherapy was not associated with cognition in non-central nervous system cancer but can play a role in patients with central nervous system cancer, Neurosurgery rather seemed to improve cognition in the short-term. These overall findings can be moderated by individual risk factors, which include brain cancer, hormone-related cancers, anti-hormonal therapy, chemotherapy, cranial radiation, genetic predisposition (e.g., APOE, COMT, BDNF), age, frailty and cognitive reserve, depression or post-traumatic distress, sleep, fatigue, and social factors. Based on the current state of the art, more research focusing on accelerated aging in older cancer patients is required to better understand the risks in subpopulations and the underlying mechanisms to improve tailored guidance and intervention studies.

## Supplementary Materials

The following supporting information can be downloaded at: https://www.mdpi.com/article/10.3390/cancers15041215/s1, Supplementary S1: Overview of the searches in the search engines; Supplementary S2: Detailed search string.

## Abbreviations

AD	Alzheimer’s disease
CRCI	Cancer-related cognitive impairment
CMF	Cyclophosphamide, methotrexate, and fluorouracil
PD	Parkinson’s disease
SES	Socioeconomic status

## Appendix A

**Table A1:** Overview of imaging studies in older cancer survivors according to the systematic literature review.

First Author, Year	Design	Cancer Type	Participants, No	Mean Age at Dx (yrs)	Mean Age at Baseline (yrs)	Measurement	Main Findings
Imaging Studies							
Ernst, 2002	CS	Breast; Other	16 Tamoxifen; 27 Healthy HRT; 33 HC	N.A.	70.4 Tamoxifen; 71.5 Estrogen; 71.8 HC	*Imaging* H MRS; MRI *Neuropsychological test* DSST; MMSE; TMT part A	- Tamoxifen or estrogen resulted in lower concentrations of myo-inositol specifically in basal ganglia - Longer duration of tamoxifen resulted in lower myo-inositol concentrations - No differences between groups on the neurocognitive tests
Nudelman, 2014	CS	All	503; 1106 HC	N.A.	71–77	*Imaging* MRI *Subjective measures* Subject, informant and clinician memory concerns; physician assessment; CDR *Neuropsychological tests* MMSE; WMS-R Logic Memory II	- Cancer history is associated with delay in AD onset independent of ApoE ε4 - NMSC was a significant driver effect - Prior cancer was associated with lower GMD in right superior frontal gyrus, driven by cancer types
Ponto, 2015	CS	Breast	10 Chemo/RT; 10 HC	>50	73.7	*Imaging* FDG PET imaging; MRI *Neuropsychological test* MMSE; ROCF; TMT part B; WMI-total	- No survivors and one HC had global PET score consistent with AD metabolic pattern - Hypometabolism in orbital frontal regions and hypermetabolism in left postcentral gyrus in survivors - Lower scores in executive functioning, working memory, and attention in survivors
Sharma, 2020	CS	Sinonasal	27 RT	N.A.	* 67	MRI	- 11% structural abnormalities in irradiated areas of brain - 63 % impaired cognitive function
Simó, 2016	PR	SCLC	11 PCI + chemo andor thoracic RT; 11 HC	N.A.	* 65	*Imaging* MRI *Neuropsychological test* AVLT; BNT; MDRS-2; ROCF; TMT part A and B; VFT (phonetic and semantic); WAIS-III Vocabulary; WAIS-III Digit Span	- Decrease in GMD in basal ganglia, (thalamus, right insula) - Decreased white matter integrity in corpus collosum - Strong association between cognitive deterioration and white matter changes in corpus collosum in survivors - 45% of survivors met criteria for cognitive impairment

**Table A2:** Overview of cognitive studies in older cancer survivors according to the systematic literature review.

Cognitive Testing							
Alibhai, 2010	PR	Prostate	77 ADT; 82 non-ADT; 82 HC	69.3 ADT 69.6 non-ADT 67.9 HC	N.A.	Animal Fluency; Card Rotations; Conditional Association Learning Test; COWAT; CVLT; Digit Span forward and backward; D-KEFS Color Word Interference Test; Judgement of Line Orientation; NART; Spatial Span forward and backward; Spatial Working Memory Task Errors; TMT part A and B	- No consistent evidence that 12 months of ADT use had adverse effect on cognitive function
Alibhai, 2017	PR	Prostate	77 ADT; 82 non-ADT; 82 HC	69.3 ADT; 69.6 non-ADT; 67.9 HC	N.A.	Animal Fluency; Card Rotations; Conditional Association Learning Test; COWAT; CVLT; Digit Span forward and backward; D-KEFS Color Word Interference Test; Judgement of Line Orientation; Spatial Span forward and backward; Spatial Working Memory Task Errors; TMT part A and B	- Ongoing use of ADT for up to 36 months was not associated with cognitive decline
Almeida, 2004	PR	Prostate	40 ADT	72.4	N.A.	CAMCO-G; WMS-III Block Design; WMS-III Verbal paired Association; WMS-III Visual Reproduction; WMS-III Word List	- Discontinuation of Tx is associated with better cognitive performance, specifically verbal memory - Chemical castration (hormonal suppression and testosterone blockade) is associated with rise in plasma levels of Aβ, and increased depression and anxiety - Verbal memory was negatively correlated with Aβ plasma levels
Alonso- Quiñones, 2020	CS	Prostate	99 ADT; 250 non-ADT; 2164 HC	N.A.	73.1	AVLT Delayed Recall; BNT; TMT part B; VFT (Semantic); WAIS-R Digital Symbol; WAIS-R Picture Completion; WAIS-R Block Design; WMS-R Logical Memory II; WMS-R Visual Reproduction II	- No association odds of mild cognitive impairment and prostate cancer, history of ADT use, including length of exposure
Alonso-Quiñones, 2021	PR	Prostate	20 ADT ≥5 years; 47 ADT <5 years; 174 non-ADT	* 70	* 78	Physician examination; Interview (Participant and informant CDR); Neuropsychological battery (9 tests)	- No association between history of ADT use and risk of mild cognitive impairment - Potential association between long-term ADT use (5+ years) and risk of mild cognitive impairment
Anstey, 2015	CS	All	81 chemo; 306 no chemo; 1562 HC;	N.A.	70.58 chemo; 70.75 no chemo; 70.58 HC	CRT; CVLT; Name as many words with letter “F” and “C”; SDMT; Spot-the-Word; SRT; TMT part B; WMS Digit Backwards	- ChemoTx at least 8 years ago is associated with faster decline in memory in late life compared to HC - ChemoTx between 1 and 8 years ago is associated with slower processing speed time compared to no chemo and HC
Buckwalter, 2005	CS	All (excluded skin) Women	541; 3123 HC	N.A.	≥74	TICS-m	- No association of cancer history with poorer cognitive function - No differences between women with or without history of cancer in delayed verbal memory
Cruzado, 2014	PR	Colon	81 pre-chemo; 73 post-chemo; 54 6-months post-chemo	66.96	N.A.	Barcelona Test Verbal Memory subtest; LMWT; SCWT; TMT part A and B; WAIS-R Digit Symbol	- Adjuvant FOLFOX4 can have negative effect on verbal memory => 54% improving, 33% worsening within 6 months after Tx - Cognitive impairment was most common in older patients and those with less years of education
Deschler, 2019	PR	Gastrointestinal; Pancreatic; Retroperitoneal sarcomas	195 surgery	N.A.	* 75	MMSE	- No cognitive decline 6 months post-surgery
Di Cristofori, 2018	PR	Meningioma	41 surgery	N.A.	* 74	Attentional Matrices Test; AVLT; Corsi Span; Digit Span Backward; Ideomotor apraxia; Naming of object; ROCF or MTCF; RPM; SCWT; Token test; VFT (phonemic); Weigl Test	- Global improvement of cognitive function between pre-surgery and 12 months post-surgery - Three patterns: fast recovery, late recovery, and progressive recovery - Fast recovery: denomination and non-verbal intelligence - Late recovery: sustained attention and constructional praxis - 50% showed deficit in visuospatial and executive functioning 12 months after surgery => longer recovery needed - At 12 months 27.4% of patients had complete cognitive recovery, 7.7% no recovery, 64.9% partial recovery
Gonzalez, 2015	PR	Prostate	58 ADT; 84 surgery only; 88 HC	N.A.	67.31 ADT; 67.72 surgery; 69.10 HC	BVMT-R (total and delayed recall); HVLT-R (total and delayed recall); NART Full-Scale IQ; SCWT; SDMT Items Completed; TIADL; WMS-III Digit Span; WMS-III Logical Memory II; WMS-III Spatial Span	- ADT resulted in impaired cognitive performance within 12 months compared to HC or prostatectomy - ADT performed at an impaired level on executive function
Hoogland, 2021	PR	Prostate	47 ADT; 82 HC	N.A.	67.6; 68.4 HC	BVMT-R (total and delayed recall); Color trails 1 and 2; COWAT; HVLT-R (total recall and delayed recall); NART Full-Scale IQ; SDMT Items Completed; TIADL; WMS-III Digit Span; WMS-III Logical Memory II; WMS-III Spatial Span	- ADT resulted in stable rates of cognitive impairment, whereas HC improved over 12 months - IL-6 levels, fatigue, and depressive symptoms increased in ADT group over 12 months - No relationship between ADT-related inflammation and cognitive impairment or depressive symptoms
Hurria, Rosen, 2006	PR	Breast	28 chemo	71	N.A.	BNT; COWAT; HVLT-R (total and delayed recall); MMSE; RCFT; SCWT; TMT part A and B; WAIS III Block Design; WAIS III Digit Symbol; WRAT-III, reading subtest	- 39% decline in cognitive function from before to 6 months after chemoTx - Most affected: visual memory, spatial function, psychomotor function, and attention
Jenkins, 2005	PR	Prostate	32 Short-term ADT + RT; 18 HC	67.5; 65.4 HC	N.A.	AVLT (supraspan and delayed); KDCT; Mental Rotation (speed and accuracy); NART; RCFT (immediate, delayed, processing speed) Semi-structured interviews; VFT (phonetic); WMS III Digit-Span task; WMS III Spatial-Span task	- After LHRH (ADT) therapy declines on tasks of spatial memory and ability - No correlation between decrease in bioavailable testosterone and cognitive performance 9 months after Tx - 25% considered their memory to be worst after 9 months of Tx
Konglund, 2013	PR	Meningioma	47 surgery	*70	N.A.	MMSE	- Improvement in cognitive function 6 months post-surgery
Kurita, 2011	CS	Breast, female gynecologic, male reproductive, all other (excluded skin and brain)	415 chemo, hormone, RT or surgery; 415 twin HC	61.9	73.3	Telephone cognitive screening (unable or poor score on cognitive screening than BDRS)	- Only female survivors were more likely to have cognitive impairment than respective twin - Increased risk of cognitive impairment in female survivors who had radiation and/or surgery, compared to chemoTx - Risk was higher among survivors of gynecologic cancers and those with Tx affecting ovarian functioning
Kvale, 2010	PR	Breast, prostate, colorectal, lymphoma, bladder, uterine, head and neck, ovarian, multiple myeloma.	37 chemo; 37 HC	N.A.	76.04; 75.81 HC	RST; TIADL; UFOV; WAIS Digit Symbol Substitution	- ChemoTx has negative impact on cognitive processing speed - Poor performance and increased age at baseline were each associated with slow processing speed post-chemoTx
La Carpia, 2020	CS	NHL	63 chemo, RT or transplant (SCT); 61 HC	N.A.	74.2; 74.3 HC	Copying drawings; Corsi Span; Digit Span; MFTC; MMSE; Nouns naming test; RAVLT; ROCF; RPM 47; SCWT; TMT part A and B; Verbs naming test; VFT (phonetic, semantic)	- Survivors had lower cognitive performance, especially in executive functioning and attention domains
Lombardi, 2018	PR	Glioblastoma	35 focal RT and chemo	≥65	N.A.	MMSE	- Lower cognitive score for survivors older than 65 years old at 9 months after RT
Mandelblatt, 2018	PR	Breast	94 chemo ± hormone; 237 hormone; 347 HC	N.A.	66.1 chemo ± hormone; 68.8 hormone; 67.8 HC	COWAT; DSST; NAB Digits (forward & backwards); NAB list A (immediate, short and long recall); Logical memory I and II; TMT Part A and B *Subjective measure* FACT-cog questionnaire	- Survivors (especially with ApoE ε4) exposed to chemoTx had lower long-term attention, processing speed and executive function and self-reported cognition scores) - Anti-hormonal Tx, with ApoE ε4 allele, resulted in short-term lower learning and memory - Self-reported cognition was associated with cognitive test - Chronological age and aging phenotypes (frailty) were associated with lower baseline attention, processing speed and executive function and self-reported cognition
Minniti, 2013	PR	Brain metastases	102 stereotactic radiosurgery	N.A.	* 77	MMSE	- No significant decline in neurocognitive function after stereotactic radiosurgery - Neurological complications (seizures, motor & speech deficits, confusion) in 13% of patients
Moon, 2014	CS	DTC	50 TSH suppression; 90 HC	N.A.	70.9 70.5 HC	*CERAD-K-N * BNT; Constructional Praxis Recall Test; Digit Span Test (backward and forward); FAB-K; MMSE; TMT part A and B; VFT; Word List (memory, recall, recognition)	- Cognitive function of older DTC survivors under long-term TSH-suppressive Tx are not impaired - Higher serum free T4 levels resulted in better scores on some cognitive domains => potential benefit of exogenous levothyroxine on cognitive function of patients who lack endogenous thyroid hormone
Morin 2018a	PR	All	403	76.15	N.A.	Total recall (Immediate and delayed)	- Three classes of cognitive functioning best fit the data: High (18%), Middle (52%) and Low recall (30%) - Stable trajectories from pre-Dx to 4 years after Dx - More depressive symptoms after Dx predicted membership to Low Recall Class - Older age, male gender, and fewer years of education predicted membership to Low Recall Class
Morin 2018b	PR	All	403 chemo, RT, surgery	76.15	N.A.	Total recall (immediate and delayed)	- None of the Tx predicted membership to the low recall class - ChemoTx predicted likely membership in the high recall class, due to age x treatment interaction. No effect surgery or RT. - Survivors <80 were more likely to receive chemoTx and have high recall cognition
Ospina-Romero, 2019	PR	All (excluded NMSC)	2250; 12,333 HC	71.7	N.A.	Immediate and delayed recall of 10-word list (proxy assessment if individual was too impaired)	- Older survivors had better memory and slower memory decline than HC - Possibility of a common pathologic process in opposite directions in cancer and AD
Paganini-Hill, 2000	PR	Breast	710 Tamoxifen; 453 No tamoxifen (other Tx)	* 60–64	* 69	Box Copying Task (Necker Cube); Clock Drawing; Narrative writing Task *Subjective measure* Survey	- Tamoxifen use for standard or longer resulted in increased physician visits for memory problems. Especially for current users - Current use of tamoxifen adversely effects cognition
Porter, 2013	CS	All (excluding skin)	1270 chemo, other Tx 8312 HC;	N.A.	74.8	Counting backwards; Naming the date, day, (vice)president; Serial 7s; Vocabulary; Word list (immediate, delayed recall, recognition) *Subjective measure* Self-rated memory	- No differences between cancer survivors and HC in cognitive function in later life - No association between chemoTx and cognition - Older age, male gender, comorbid conditions, depressive symptoms, less education associated with worse condition
Regier, 2019	PR	Oral-digestive Males	88 surgery, chemo, RT; 88 HC	N.A.	65.93; 72.85 HC	MoCA	- 40% survivors had cognitive impairment 18 months post-Dx - At 18 months, cognition improved in comparison to 6 months (improvement in phonemic fluency and memory) - Neither chemo or RT associated with worse cognition - Older age, low hemoglobin, and cancer-related PTSD were associated with worse cognition at 18 months
Shaffer, 2012	PR	Breast and colorectal	24 breast chemo; 64 colo chemo; 117 breast no chemo; 160 colo no chemo	75.5	N.A.	TICS-m	- No differences in rates of cognitive decline before and after Dx - Cognitive decline after Dx did not differ between those receiving chemoTx and those who did not
Schilder, 2010	PR	Breast	80 Tamoxifen; 99 Exemestane; 120 HC	N.A.	68.7 Tamoxifen; 68.3 Exemestane; 66.2 HC	Fepsy Finger Tapping; Fepsy Reaction Time; RAVLT; SCWT; TMT part A and B; VFT (phonetic, semantic); Visual Association Test; WAIS-III Letter-Number Sequencing; WMS-R Visual Memory	- 1 year of tamoxifen is associated with lower functioning in verbal memory and executive functioning - 1 year of exemestane is not associated with negative effects on cognitive functioning - Processing speed was worse for tamoxifen group compared to exemestane group - Older age tamoxifen group resulted in worse verbal memory and processing speed compared to HC - Younger age tamoxifen group performed worse on executive functioning compared to HC
Tan, 2013	PR	Prostate	50 ADT	* 71	N.A.	California Verbal Learning Test-Short Form; MMSE	- No cognitive decline or change in memory function over time after Tx with leuprolide - No elevated plasma amyloid short term after Tx with leuprolide - Age was correlated with plasma amyloid Aβlevels
Underwood, 2019	PR	Breast	42 hormone	N.A.	68.38	BVMT-R; RAVLT; TMT part A and B; VFT (phonetic); WAIS-IV Digit Symbol Coding; WAIS-IV Symbol Search; WAIS-IV Matrix Reasoning; WAIS-IV Block Design;; WAIS-IV Visual Puzzles	- Decline on verbal memory in older women after 1 year of endocrine therapy (ET)
van der Willik, 2021	PR	Non-CNS	718 No Tx, local Tx, chemo or hormone; 4859 HC	* 70.3	N.A.	LDST; MMSE; PPT; SCWT; WFT; WLT (immediate, delayed recall and recognition)	- Cognitive trajectories of cancer survivors were similar to HC (but most received local therapy only) - After Dx largest decline was found in memory
Williams, 2016	CS	All (excluding skin)	408; 2639 HC	N.A.	72.87; 70.67 HC	DSST; Self-reported memory of confusion problems	- Cancer survivors have more cognitive impairment (processing speed, attention, executive function, learning and working memory) - 17% higher odds of self-reported problems with memory or confusion in cancer survivors
Yang, 2015	CS	Prostate	43 ADT; 35 non-ADT; 40 HC	N.A.	69.28 ADT; 68.83 non-ADT; 67.80 HC	AVLT (immediate, delayed recall, recognition); MoCA; SCWT; TMT A and B; VFT; WAIS III-R Digit Span (forward & backward)	- ADT group had lower scores on cognitive tasks including attention, memory and information processing - Receiving ADT may have selective reductions in EBPM performance but unimpaired TBPM performance - Deficits induced by ADT may result from changes in function and structure of the pre-frontal cortex
Yamada, 2010	CS	Breast	30 chemo; 30 HC	>50	72.8; 72.6 HC	BNT; BVRT; COWAT; Facial Recognition Test; IED Stage 5 errors; MMSE; RAVLT; ROCF; TMT part A and B; WAIS-III Digit Span; WAIS-III Letter-number Sequencing; WAIS-III Arithmetic subtests; WASI; WCST; WRAT-III reading subtest	- In long-term survivors, previous Tx may augment cognitive dysfunction associated with age-related brain changes - Domains affected: global cognition, attention, working memory, psychomotor speed, and executive functioning - Reflects potential dysfunction in frontal-subcortical brain region

**Table A3:** Overview of self-report studies in older cancer survivors according to the systematic literature review.

Questionnnaires/Interviews							
Freedman, 2013	PR	Breast	297 chemo	* 71.5	N.A.	NBF-ADL	- ChemoTx was not associated with longitudinal changes in self-reported cognitive function - No changes by Tx (standard vs capecitabine) - At 24 months, most survivors reported normal cognition
Heflin, 2005	CS	All (excluded brain)	702; 702 twin HC	N.A.	74.9	Telephone cognitive screening (unable or poor score on cognitive screening then BDRS); If score 3 Dementia screening using Diagnostic and Statistical Manual-IV criteria	- Long-term survivors presented higher rates of cognitive dysfunction complaints than their healthy twin - Cancer survivors were twice as likely to be diagnosed with dementia as their healthy twin
Hurria, Goldfarb, 2006	PR	Breast	45 chemo	70	N.A.	Squire Memory Self-Rating Questionnaire	- 51% of survivors perceived decline in memory when comparing to before chemoTx
Keating, 2005	CS	All	964; 14 330 HC	55.0	68.3	Survey TICS	- Similar rates of self-reported memory, and among 65+, similar cognition scores between survivors and HC - No difference in cognitive complaints between survivors and HC
Mandelblatt, 2016	PR	Breast	519 chemo ± hormone 687 hormone alone	N.A.	72.7	EORTC-QLQ-C30	- Most older survivors maintained good long-term self-reported cognitive function (42%) - 2.1x higher odds of accelerated decline when exposed to chemoTx (with or without anti-hormonal Tx) - Comorbidity (or frailty) and low pre-diagnosis function increased odds of accelerated cognitive decline - Age was not related to accelerated group - Cognition trajectories were related to physical function trajectories
Schilder, 2012	PR	Breast	80 Tamoxifen; 99 Exemestane; 120 HC	N.A.	68.7 Tamoxifen; 68.3 Exemestane; 66.2 HC	CFQ Dutch version; Interview questions “Do you have any complaints with regard to memory and attention/ concentration?” *Neuropsychological tests* Dutch Adult Reading Test; Fepsy Finger Tapping; Fepsy Reaction Times; RAVLT (immediate and delayed); SCWT; TMT A and B; VFT (phonetic, semantic); Visual Association Test; WAIS-III Letter-Number sequencing; WMS-R Visual Memory (immediate and delayed)	- Increased attention/concentration complaints in tamoxifen users 1 year after Tx, but not in exemestane - Tamoxifen or exemestane use did not influence self-reported frequency of cognitive failures - Self-reported cognitive function was associated with anxiety/depression, fatigue, and menopausal complaints - Cognitive test was not associated with self-reported cognitive functioning
Stava, 2006	CS	Breast; Other Female	814 breast cancer; - 334 chemo; - 470 no chemo 1894 other cancers	46.8 breast; 42.7 other	69.4 breast; 66.4 other	Survey	- Survivors who received chemoTx more frequently reported loss of memory (11.3% vs. 4.8%) - Women who received chemoTx more frequently reported loss of memory (12.3% vs. 7.2%) - Breast cancer survivors reported more loss of memory than other cancers (18.4% vs. 12.5%)

NOTE. * Indicates median. Abbreviations: 3MS, Modified Mini-Mental State Examination; AD, Alzheimer’s Disease; ADT, Androgen Deprivation Therapy; ApoE ε4, Apolipoprotein E; AVLT, Auditory Verbal Learning Test; BDRS, Blessed Dementia Rating Scale; BNT, Boston Naming Test; BVMT-R, Brief Visual-Memory Test-Revised; BVRT, Benton Visual Retention Test; CAMCO-G, Cambridge Cognitive Examination; CDR, Clinical Dementia Rating; CERAD-K-N, Consortium to Establish a Registry for Alzheimer’s Disease; CFQ, Cognitive Failure Questionnaire; CNS, Central Nervous System; COWAT, Controlled Oral Word Association Test; CRT, Complex reaction time; CS, Cross-sectional; CVLT, California Verbal Learning Test; D-KEFS, Delis-Kaplan Executive Function System; DSST, Digit Symbol Substitution Test; DTC, differentiated thyroid cancers; Dx, Diagnosis; EBPM, event-based prospective memory; EORTC-QLQ-C30, European Organization for Research and Treatment for Cancer Quality of Life Questionnaire; FAB-K, Frontal Assessment Battery; FACT-cog, Functional Assessment of Cancer Therapy-Cognitive Function; FDG, fluorodeoxyglucose; GMD, gray matter density; HC, healthy controls; H MRS, Proton Magnetic Resonance Spectroscopy; HVLT-R, Hopkins Verbal Learning Test, Revised; IED; Intradimensional/Extradimensional; IL-6, Interleukin 6; KDCT, Kendrick Assessment of Cognitive Aging battery; LDST, Letter-Digit Substitution Test; LHRH, Luteinizing hormone releasing hormone; LMWT, Luria Memory Word test; MDRS-2, Mattis Dementia Rating Scale-2; MFTC, Multiple Features Target Cancellation Test; MMSE, Mini-Mental State Exam; MoCA, Montreal Cognitive Assessment; MRI, Magnetic Resonance Imaging; MTCF, Modified Taylor Complex Figure Test; NAB, Neuropsychological Assessment Battery; NART, National Adult Reading Test; NBFADL, Neurobehavioral Functioning and Activities of Daily Living Scale; NHL, Non-Hodgkin Lymphoma; NMSC, Non-Melanoma Skin Cancer; No, number; PCI, prophylactic cranial irradiation; PET, Positron emission tomography; PMA-V, Primary Mental Abilities-Vocabulary; PPT, Purdue Pegboard Test; PR, Prospective; PTSD, Post-Traumatic Stress Disorder; RAVLT, Rey Auditory Verbal Learning Test; RCFT, Rey Complex Figure Test; ROCF, Rey–Osterrieth Complex Figure; RPM, Raven’s Progressive Matrices; RST, The Road Sign Test; RT, Radiotherapy; SCLC, Small Cell lung Cancer; SCWT, Stroop Color Word Test; SDMT, Symbol Digit Modalities Test; SRT, Simple reaction time; TBPM, time-based prospective memory; TIADL, Timed Instrumental Activities of Daily Living; TICS-m, Telephone interview cognitive screening; TMT, Trail Making Test; TSH, Thyroid Stimulating Hormone; Tx, Treatment; UFOV, Useful Field Of Vision; VFT, Verbal Fluency Test; WAIS-III, Wechsler Adult Intelligence Scale III; WASI, Wechsler Abbreviated Scale of Intelligence; WCST, Wisconsin Card Sorting Test; WFT, Word Fluency Test; WLT, 15-Word Learning Test; WMI, Working Memory Index; WMS, Wechsler Memory Scale; WRAT, Wide Range Achievement Test; Yrs, years.

## Appendix B

**Table A4:** Overview of neurodegenerative diseases in older cancer survivors according to the systematic literature review.

First Author/Year	Cancer Subtype	Study Participants and Controls, No	Disease Outcome	Main Findings
Baik, 2017	Prostate	440,129 ADT; 798,750 non-ADT	AD; Dementia	- No association between ADT use and AD or dementia
Baxter, 2009	Breast	2913 chemo; 18,449 no chemo	Senile dementia; Presenile dementia; Drug-induced dementia; AD; Pick’s disease; Senile degeneration of the brain; Other cerebral degeneration; Toxic encephalopathy; Senility	- ChemoTx was not associated with greater risk of dementia Dx over time - Hormone receptor status was not associated with dementia or possible dementia Dx
Blanchette, 2020	Breast	8770 AI; 3307 Tamoxifen	Dementia	- AI Tx was associated with decreased incidence of dementia compared tamoxifen Tx
Boulet, 2019	Thorax, head and neck	5718 RT - 4166 statin use - 1552 nonusers	Cerebrovascular events: -Transient ischemic attack; -Stroke; -Carotid revascularization; -Stroke death	- Statin use post RT was associated with reduction in stroke, with a trend toward reducing cerebrovascular events
Bowles, 2017	All	756 prevalent cancer; 583 incident cancer	Dementia; Possible AD; Probable AD	- Prevalent cancer was not associated with decreased risk of dementia or AD - Incident cancer was associated with decreased risk of AD
Branigan, 2020	Breast	18,126 hormone Tx; 39,717 no hormone Tx	AD; MS; PD; ALS	- Tamoxifen and steroid-AI were associated with a decrease in Dx of NDD, specifically AD and dementia
Bromley, 2019	Breast	8018 AI; 6296 Tamoxifen	AD; VaD; Dementia with Lewy bodies; Mixed dementia; Unknown type	- No evidence for a difference in dementia, AD or VaD risk between AI and tamoxifen users
Chen, 2011	Lung	52,089; 104,178 HC	Stroke	- Lung cancer was associated with increased risk of subsequent stroke within 1 year after Dx for men and 2 years for women - Risk was stronger for hemorrhagic than for ischemic stroke
Chung, 2016	Prostate	1335 ADT; 4005 HC	AD; PD	- ADT use was not associated with higher risk of AD or PD
Driver, 2012	All (excluded NMSC)	176; 1102 HC	Any dementia; Possible AD; Probable AD	- Cancer survivors had a lower risk of AD - Risk of AD was lowest in smoking related cancers
Du, 2013	Colorectal	23,484 chemo; 48,890 no chemo	Unspecified cognitive disorder; Amnestic disorder; AD; VaD; Unspecified dementia; Drug-induced dementia; Psychoses	- ChemoTx use 24% more likely to develop drug-induced dementia - Risk of AD, VaD or other dementias was lower when receiving chemoTx - Cognitive disorder was not different between groups
Du, 2010	Breast	14,057 chemo; 48,508 no chemo	Cognitive disorder NOS; Amnestic disorder; AD; VaD; Dementia; Drug induced dementia; Psychoses	- No association between chemoTx and risk of developing drug-induced dementia & unspecified cognitive disorders - Risk of developing AD, VaD, or other dementias was lower in patients receiving chemoTx - Cognitive disorder was not different between groups
Elbaz, 2002	All	38; 46 HC	PD	- No strong association between nonfatal cancer and PD - Suggestive trends stratified by sex and age at onset of PD, and for specific cancers related to smoking or hormonal factors
Fowler, 2020	Breast, prostate, colorectal, NMSC	36 breast; 103 prostate; 29 colorectal; 165 NMSC; 904 HC	Progression of AD	- Cancer history associated with better cognition and later age of AD onset - Progression was similar to those without cancer history
Frain, 2017	All (excluded NMSC)	771,285; 2,728,093 HC	Alzheimer’s disease; Non-AD dementia; Stroke; Macular degeneration.	- Non-screening-related cancer survivors had lower AD risk - Screening-related cancer survivors had increased AD risk - ChemoTx was associated with decreased risk of AD
Freedman, 2016	All	836,947; 142,869 HC	Alzheimer’s disease	- Risk of AD was 13% lower in cancer patients
Hanson, 2017	All	92,425	AD	- Cancer was not protective against AD - Under certain model specifications, inverse association between cancer and AD can be replicated
Heck, 2008	Breast	6289 chemo 12,071 no chemo	Dementia	- Increase in dementia Dx over time (long-term) in subjects who received chemoTx
Hong, 2013	Glottic	358 surgery; 1055 RT	Cerebrovascular events	- Early-stage laryngeal cancer post-surgery or RT have a higher burden of cerebrovascular events - RT and surgery are associated with comparable risk of subsequent CVD after Tx.
Hong, 2020	Prostate	12,740 ADT; 4685 no ADT	Overall cognitive decline; Dementia (including AD and non-AZD); PD	- Higher risk of overall cognitive dysfunction in patients receiving ADT - Antiandrogen-only ADT was associated with increased risks of overall cognitive decline, PD, and non-AZD, but not AD - CAB was associated with higher risks of overall cognitive decline and non-AD. - ADT duration did not correlate with cognitive dysfunction
Huang, 2020	Prostate	6904 no ADT; 11,817 GnRH; 876 orchiectomy; 4054 anti-androgen monotherapy	Dementia; AD	- Use of antiandrogen monotherapy was associated with increased risk of dementia or AD - GnRH agonist use and orchiectomy had no difference compared with patients who did not receive ADT.
Ibler, 2018	Melanoma and NMSC	1147 MM; 2506 BCC; 967 SCC; 78,305 controls	AD	- Decreased risk for AD in patients with MM and NMSC
Jayadevappa, 2019	Prostate	62,330 ADT; 91,759 no ADT	Dementia; AD	- Use of ADT was associated with subsequent Dx of dementia or AD over a follow-up period of 10 years
Jazzar, 2020	Bladder	2403 RC 2411 RT andor CTX	AD; Related dementias	- No difference in new-onset AD and related dementias after surgery, RT or chemoTx (risk similar regardless of Tx) - Older patients and patients with depression were at increased risk of developing dementia related diseases
Jhan, 2017	Prostate	15,959 ADT; 8401 no ADT	AD	- ADT use is associated with increased risk of developing AD
Kao, 2015	Prostate	755 ADT; 559 no ADT	Dementia	- No difference in risk of subsequent dementia in ADT receivers versus no-ADT receivers - No association between GnRH agonists and incidence of dementia in prostate cancer survivors
Khan, 2011	Breast, colorectal and prostate	26,213; 104,486 HC	Dementia	- Increased incidence of dementia in colorectal cancer survivors compared to controls
Khosrow-Khavar, 2017	Prostate	15,310 ADT; 15,593 no ADT	All dementia events, including AD	- ADT use was not associated with increased risk of dementia
Klinger, 2021	Bladder	1587 BCG; 5147 non-BCG	New-onset dementia; New-onset AD; PD; Stroke	- BCG Tx in bladder cancer was associated with reduced risk of AD (and PD)
Krasnova, 2020	Prostate	37,911 ADT; 62,503 no ADT	AD; All-cause dementia	- Association between pharmacologic ADT and higher risk of all-cause dementia, AD, and use of psychiatric services
Liao, 2017	Breast	173 breast with AD; 684 breast no AD	AD	- Increased odds of AD associated with ever use tamoxifen - Longer tamoxifen use was associated with increased AD (survival effect)
Mahajan, 2020	Skin	125; 125 HC	PD	- Skin cancer, may delay onset but not progression of PD
Musicco, 2013	All	21,451	AD	- Inverse association between cancer and AD - Older people with cancer have a reduced risk of AD
Nead, 2016	Prostate	2397 ADT; 14,491 no ADT	New-onset AD	- Use of ADT resulted in an increased future risk of AD
Nead, 2017	Prostate	1826 ADT; 7446 no ADT	New-onset dementia	- Use of ADT was associated with an increased risk of dementia - Longer ADT use resulted in increased risk of dementia
Olsen, 2006	All	8090 PD; 32,320 control	PD	- Increased prevalence of malignant melanoma and skin carcinoma before Dx of PD - Decreased prevalence of smoking-related cancers before PD
Ording, 2020	All	679,122; 3,395,597 HC	AD; VaD, All-cause dementia	- No clinically relevant association between cancer and risk of AD, Vad, or all-cause dementia
Raji, 2009	Breast	6932 Anthracycline, CMF, Taxane, others	Dementia	- No association was found between types of chemoTx agents for breast cancer and risk of new dementia Dx - Increasing age at cancer Dx, black ethnicity, living in census tract with level of lower education, and increasing number of comorbidities associated with new dementia Dx.
Realmuto, 2012	All	126 AD; 252 HC	AD	- Inverse association between AD and cancer diagnosed before onset of dementia (limited to endocrine-related tumors)
Roe, 2010	All	3020	Any AD; Pure AD; Any VaD; Pure VaD; Mixed AD/VaD	- Cancer history was associated with reduced risk of any or pure AD - No association between cancer history and any or pure VaD, or mixed dementia
Roderburg, 2021	All	92,868; 92,868 HC	Dementia; Mild cognitive impairment	- Increased incidence of dementia in patients with different cancer entities (19.7%) than in non-cancer patients (16.7%) - Effect most pronounced in lung cancer patients
Shahinian, 2006	Prostate	5748 ADT; 34,865 no ADT; 50,476 HC	Senile Dementia; Organic or Drug-related memory disturbances; Cerebral degenerations; Any cognitive disorder	- Cognitive disorders were increased in patients receiving ADT, due to increased age, comorbid conditions, and more advanced cancers
Smith, 2009	Head and neck	1983 RT; 2823 surgery + RT; 2056 surgery	Cerebrovascular events: -Stroke; Carotid revascularization; Stroke death	- RT for head and neck cancer, but not postoperative RT, was associated with cerebrovascular disease risk in older patients
Sun, 2020	35 types	732,901; 1,769,357 HC	Dementia	- Risk of dementia was lower up to 10 years of follow up - Risk of dementia was higher after more than 10 years survival
Tae, Jeon, Shin 2019	Prostate	24,929 ADT; 12,620 no ADT	Cognitive dysfunction: - Dementia; AD	- ADT use was associated with increased risk of cognitive dysfunction
Tae, Jeon, Choi 2019	Prostate	24,069 ADT; 12,077 no ADT	Cerebral infarction	- ADT was not associated with cerebral infarction
White 2013	NMSC	1102	Dementia; Possible AD; Probable AD; Mixed VaD	- NMSC had reduced risk of developing AD
Zhu, 2015	All	322,558; 5,365,608 HC	Transient global amnesia (TGA)	- No evidence that patients with cancer had higher risk of TGA than cancer-free individuals

NOTE. Abbreviations: AD, Alzheimer’s Disease; ADT, Androgen Deprivation Therapy; AI, Aromatase inhibitors; ALS, Lou Gehrig’s disease; BCC, basal cell cancers; BCG, Bacillus Calmette–Guérin vaccine; CAB, Combined Androgen Blockage; CMF, Cyclofosfamide Methotrexaat Fluorouracil; CTX: cyclophosphamide chemotherapy; CVD, Cerebral Vascular Disease; Dx, Diagnosis; GnRH, Gonadotropin-releasing Hormone; HC, Healthy Controls; MM, Multiple Melanoma; MS, multiple sclerosis; NMSC, Non-Melanoma Skin Cancer; NOS, Not Otherwise Specified; PD, Parkinson’s Disease; RC, radical cystectomy; RT, Radiotherapy; SCC, squamous cell cancers; VaD, Vascular Dementia.

## References

[1] Sung H., Ferlay J., Siegel R.L., Laversanne M., Soerjomataram I., Jemal A., Bray F. (2021). Global Cancer Statistics 2020: GLOBOCAN Estimates of Incidence and Mortality Worldwide for 36 Cancers in 185 Countries. CA Cancer J. Clin..

[2] Henderson T.O., Ness K.K., Cohen H.J. (2014). Accelerated Aging among Cancer Survivors: From Pediatrics to Geriatrics. Am. Soc. Clin. Oncol. Educ. Book.

[3] Lange M., Rigal O., Clarisse B., Giffard B., Sevin E., Barillet M., Eustache F., Joly F. (2014). Cognitive Dysfunctions in Elderly Cancer Patients: A New Challenge for Oncologists. Cancer Treat. Rev..

[4] Small B.J., Scott S.B., Jim H.S.L., Jacobsen P.B. (2015). Is Cancer a Risk Factor for Cognitive Decline in Late Life?. Gerontology.

[5] Lange M., Heutte N., Noal S., Rigal O., Kurtz J.-E., Lévy C., Allouache D., Rieux C., Lefel J., Clarisse B. (2019). Cognitive Changes After Adjuvant Treatment in Older Adults with Early-Stage Breast Cancer. Oncologist.

[6] Janelsins M.C., Kesler S.R., Ahles T.A., Morrow G.R. (2014). Prevalence, Mechanisms, and Management of Cancer-Related Cognitive Impairment. Int. Rev. Psychiatry.

[7] Ahles T.A., Saykin A.J. (2007). Candidate Mechanisms for Chemotherapy-Induced Cognitive Changes. Nat. Rev. Cancer.

[8] Makale M.T., McDonald C.R., Hattangadi-Gluth J.A., Kesari S. (2017). Mechanisms of Radiotherapy-Associated Cognitive Disability in Patients with Brain Tumours. Nat. Rev. Neurol..

[9] Rogiers A., Boekhout A., Schwarze J.K., Awada G., Blank C.U., Neyns B., Gupta S.C. (2019). Long-Term Survival, Quality of Life, and Psychosocial Outcomes in Advanced Melanoma Patients Treated with Immune Checkpoint Inhibitors. J. Oncol..

[10] Joly F., Castel H., Tron L., Lange M., Vardy J. (2020). Potential Effect of Immunotherapy Agents on Cognitive Function in Cancer Patients. J. Natl. Cancer Inst..

[11] Vannorsdall T.D. (2017). Cognitive Changes Related to Cancer Therapy. Med. Clin. N. Am..

[12] Ahles T.A., Hurria A. (2018). New Challenges in Psycho-Oncology Research IV: Cognition and cancer: Conceptual and methodological issues and future directions. Psycho-Oncology.

[13] Ahles T.A., Root J.C. (2018). Cognitive Effects of Cancer and Cancer Treatments. Annu. Rev. Clin. Psychol..

[14] Hardy S.J., Krull K.R., Wefel J.S., Janelsins M. (2018). Cognitive Changes in Cancer Survivors. Am. Soc. Clin. Oncol. Educ. Book.

[15] M Steverson Ageing and Health. https://www.who.int/news-room/fact-sheets/detail/ageing-and-health#:~:text=At%20the%20biological%20level%2C%20ageing,of%20disease%20and%20ultimately%20death.

[16] Muhandiramge J., Orchard S., Haydon A., Zalcberg J. (2021). The Acceleration of Ageing in Older Patients with Cancer. J. Geriatr. Oncol..

[17] Mandelblatt J.S., Jacobsen P.B., Ahles T. (2014). Cognitive Effects of Cancer Systemic Therapy: Implications for the Care of Older Patients and Survivors. J. Clin. Oncol..

[18] Nudelman K.N.H., Risacher S.L., West J.D., McDonald B.C., Gao S., Saykin A.J. (2014). Association of Cancer History with Alzheimer’s Disease Onset and Structural Brain Changes. Front. Physiol..

[19] Sharma M.B., Jensen K., Urbak S.F., Funding M., Johansen J., Bechtold D., Amidi A., Eskildsen S.F., Jørgensen J.O.L., Grau C. (2020). A Multidimensional Cohort Study of Late Toxicity after Intensity Modulated Radiotherapy for Sinonasal Cancer. Radiother. Oncol..

[20] Simó M., Vaquero L., Ripollés P., Jové J., Fuentes R., Cardenal F., Rodríguez-Fornells A., Bruna J. (2016). Brain Damage Following Prophylactic Cranial Irradiation in Lung Cancer Survivors. Brain Imaging Behav..

[21] Ponto L.L.B., Menda Y., Magnotta V.A., Yamada T.H., Denburg N.L., Schultz S.K. (2015). Frontal Hypometabolism in Elderly Breast Cancer Survivors Determined by [18F]Fluorodeoxyglucose (FDG) Positron Emission Tomography (PET): A Pilot Study. Int. J. Geriatr. Psychiatry.

[22] Mandelblatt J.S., Hurria A., McDonald B.C., Saykin A.J., Stern R.A., Vanmeter J.W., McGuckin M., Traina T., Denduluri N., Turner S. (2013). Cognitive Effects of Cancer and Its Treatments at the Intersection of Aging: What Do We Know; What Do We Need to Know?. Semin. Oncol..

[23] Ernst T., Chang L., Cooray D., Salvador C., Jovicich J., Walot I., Boone K., Chlebowski R. (2002). The Effects of Tamoxifen and Estrogen on Brain Metabolism in Elderly Women. J. Natl. Cancer Inst..

[24] Yamada T.H., Denburg N.L., Beglinger L.J., Schultz S.K. (2010). Neuropsychological Outcomes of Older Breast Cancer Survivors: Cognitive Features Ten or More Years after Chemotherapy. J. Neuropsychiatry Clin. Neurosci..

[25] Di Cristofori A., Zarino B., Bertani G., Locatelli M., Rampini P., Carrabba G., Caroli M. (2018). Surgery in Elderly Patients with Intracranial Meningioma: Neuropsychological Functioning during a Long Term Follow-Up. J. Neurooncol..

[26] Konglund A., Rogne S.G., Lund-Johansen M., Scheie D., Helseth E., Meling T.R. (2013). Outcome Following Surgery for Intracranial Meningiomas in the Aging. Acta Neurol. Scand..

[27] Minniti G., Esposito V., Clarke E., Scaringi C., Bozzao A., Lanzetta G., de Sanctis V., Valeriani M., Osti M., Enrici R.M. (2013). Stereotactic Radiosurgery in Elderly Patients with Brain Metastases. J. Neurooncol..

[28] Lombardi G., Bergo E., Bianco P.D., Bellu L., Pambuku A., Caccese M., Trentin L., Zagonel V. (2018). Quality of Life Perception, Cognitive Function, and Psychological Status in a Real-World Population of Glioblastoma Patients Treated with Radiotherapy and Temozolomide: A Single-Center Prospective Study. Am. J. Clin. Oncol..

[29] Deschler B., Ihorst G., Hüll M., Baier P. (2019). Regeneration of Older Patients after Oncologic Surgery. A Temporal Trajectory of Geriatric Assessment and Quality of Life Parameters. J. Geriatr. Oncol..

[30] Gonzalez B.D., Jim H.S.L., Booth-Jones M., Small B.J., Sutton S.K., Lin H.Y., Park J.Y., Spiess P.E., Fishman M.N., Jacobsen P.B. (2015). Course and Predictors of Cognitive Function in Patients With Prostate Cancer Receiving Androgen-Deprivation Therapy: A Controlled Comparison. J. Clin. Oncol..

[31] Porter K.E. (2013). “Chemo Brain”-Is Cancer Survivorship Related to Later-Life Cognition? Findings from the Health and Retirement Study. J. Aging Health.

[32] Morin R.T., Midlarsky E. (2018). Treatment with Chemotherapy and Cognitive Functioning in Older Adult Cancer Survivors. Arch. Phys. Med. Rehabil..

[33] Regier N.G., Naik A.D., Mulligan E.A., Nasreddine Z.S., Driver J.A., Sada Y.H.F., Moye J. (2019). Cancer-Related Cognitive Impairment and Associated Factors in a Sample of Older Male Oral-Digestive Cancer Survivors. Psychooncology.

[34] Van der Willik K.D., Jóźwiak K., Hauptmann M., van de Velde E.E.D., Compter A., Ruiter R., Stricker B.H., Ikram M.A., Schagen S.B. (2021). Change in Cognition before and after Non-Central Nervous System Cancer Diagnosis: A Population-Based Cohort Study. Psychooncology.

[35] Kurita K., Meyerowitz B.E., Hall P., Gatz M. (2011). Long-Term Cognitive Impairment in Older Adult Twins Discordant for Gynecologic Cancer Treatment. J. Gerontol. A Biol. Sci. Med. Sci..

[36] Mandelblatt J.S., Small B.J., Luta G., Hurria A., Jim H., McDonald B.C., Graham D., Zhou X., Clapp J., Zhai W. (2018). Cancer-Related Cognitive Outcomes Among Older Breast Cancer Survivors in the Thinking and Living with Cancer Study. J. Clin. Oncol..

[37] Schilder C.M., Seynaeve C., Beex L.V., Boogerd W., Linn S.C., Gundy C.M., Huizenga H.M., Nortier J.W., van de Velde C.J., van Dam F.S. (2010). Effects of Tamoxifen and Exemestane on Cognitive Functioning of Postmenopausal Patients with Breast Cancer: Results from the Neuropsychological Side Study of the Tamoxifen and Exemestane Adjuvant Multinational Trial. J. Clin. Oncol..

[38] Paganini-Hill A., Clark L.J. (2000). Preliminary Assessment of Cognitive Function in Breast Cancer Patients Treated with Tamoxifen. Breast Cancer Res. Treat..

[39] Underwood E.A., Jerzak K.J., Lebovic G., Rochon P.A., Elser C., Pritchard K.I., Tierney M.C. (2019). Cognitive Effects of Adjuvant Endocrine Therapy in Older Women Treated for Early-Stage Breast Cancer: A 1-Year Longitudinal Study. Support. Care Cancer.

[40] Almeida O.P., Waterreus A., Spry N., Flicker L., Martins R.N. (2004). One Year Follow-up Study of the Association between Chemical Castration, Sex Hormones, Beta-Amyloid, Memory and Depression in Men. Psychoneuroendocrinology.

[41] Hoogland A.I., Jim H.S.L., Gonzalez B.D., Small B.J., Gilvary D., Breen E.C., Bower J.E., Fishman M., Zachariah B., Jacobsen P.B. (2021). Systemic Inflammation and Symptomatology in Patients with Prostate Cancer Treated with Androgen Deprivation Therapy: Preliminary Findings. Cancer.

[42] Jenkins V.A., Bloomfield D.J., Shilling V.M., Edginton T.L. (2005). Does Neoadjuvant Hormone Therapy for Early Prostate Cancer Affect Cognition? Results from a Pilot Study. BJU Int..

[43] Yang J., Zhong F., Qiu J., Cheng H., Wang K. (2015). Dissociation of Event-Based Prospective Memory and Time-Based Prospective Memory in Patients with Prostate Cancer Receiving Androgen-Deprivation Therapy: A Neuropsychological Study. Eur. J. Cancer Care.

[44] Alibhai S.M.H., Breunis H., Timilshina N., Marzouk S., Stewart D., Tannock I., Naglie G., Tomlinson G., Fleshner N., Krahn M. (2010). Impact of Androgen-Deprivation Therapy on Cognitive Function in Men with Nonmetastatic Prostate Cancer. J. Clin. Oncol..

[45] Alibhai S.M.H., Timilshina N., Duff-Canning S., Breunis H., Tannock I.F., Naglie G., Fleshner N.E., Krahn M.D., Warde P., Marzouk S. (2017). Effects of Long-Term Androgen Deprivation Therapy on Cognitive Function over 36 Months in Men with Prostate Cancer. Cancer.

[46] Alonso-Quiñones H., Stish B.J., Aakre J.A., Hagen C.E., Petersen R.C., Mielke M.M. (2021). Androgen Deprivation Therapy Use and Risk of Mild Cognitive Impairment in Prostate Cancer Patients. Alzheimer Dis. Assoc. Disord..

[47] Alonso Quiñones H.J., Stish B.J., Hagen C., Petersen R.C., Mielke M.M. (2020). Prostate Cancer, Use of Androgen Deprivation Therapy, and Cognitive Impairment: A Population-Based Study. Alzheimer Dis. Assoc. Disord.

[48] Tan W.W., Heckman M.G., Vishnu P., Crook J.E., Younkin L.H., Covil E.G., Ferman T.J., Graff-Radford N.R., Younkin S.G., Smallridge R.C. (2013). Effect of Leuprolide on Serum Amyloid-β Peptide Levels and Memory in Patients with Prostate Cancer with Biochemical Recurrence. Urology.

[49] Moon J.H., Ahn S., Seo J., Han J.W., Kim K.M., Choi S.H., Lim S., Park Y.J., Park D.J., Kim K.W. (2014). The Effect of Long-Term Thyroid-Stimulating Hormone Suppressive Therapy on the Cognitive Function of Elderly Patients with Differentiated Thyroid Carcinoma. J. Clin. Endocrinol. Metab..

[50] Anstey K.J., Sargent-Cox K., Cherbuin N., Sachdev P.S. (2015). Self-Reported History of Chemotherapy and Cognitive Decline in Adults Aged 60 and Older: The PATH Through Life Project. Med. Sci..

[51] Cruzado J.A., López-Santiago S., Martínez-Marín V., José-Moreno G., Custodio A.B., Feliu J. (2014). Longitudinal Study of Cognitive Dysfunctions Induced by Adjuvant Chemotherapy in Colon Cancer Patients. Support Care Cancer.

[52] Hurria A., Rosen C., Hudis C., Zuckerman E., Panageas K.S., Lachs M.S., Witmer M., van Gorp W.G., Fornier M., D’Andrea G. (2006). Cognitive Function of Older Patients Receiving Adjuvant Chemotherapy for Breast Cancer: A Pilot Prospective Longitudinal Study. J. Am. Geriatr. Soc..

[53] Kvale E.A., Clay O.J., Ross-Meadows L.A., McGee J.S., Edwards J.D., Unverzagt F.W., Ritchie C.S., Ball K.K. (2010). Cognitive Speed of Processing and Functional Declines in Older Cancer Survivors: An Analysis of Data from the ACTIVE Trial. Eur. J. Cancer Care.

[54] Shaffer V.A., Merkle E.C., Fagerlin A., Griggs J.J., Langa K.M., Iwashyna T.J. (2012). Chemotherapy Was Not Associated with Cognitive Decline in Older Adults with Breast and Colorectal Cancer: Findings from a Prospective Cohort Study. Med. Care.

[55] Buckwalter J.G., Crooks V.C., Petitti D.B. (2005). Cognitive Performance of Older Women Who Have Survived Cancer. Int. J. Neurosci..

[56] Morin R.T., Midlarsky E. (2018). Depressive Symptoms and Cognitive Functioning among Older Adults with Cancer. Aging Ment. Health.

[57] La Carpia D., Liperoti R., Guglielmo M., di Capua B., Devizzi L.F., Matteucci P., Farina L., Fusco D., Colloca G., di Pede P. (2020). Cognitive Decline in Older Long-Term Survivors from Non-Hodgkin Lymphoma: A Multicenter Cross-Sectional Study. J. Geriatr. Oncol..

[58] Williams A.M., Janelsins M.C., van Wijngaarden E. (2016). Cognitive Function in Cancer Survivors: Analysis of the 1999-2002 National Health and Nutrition Examination Survey. Support Care Cancer.

[59] Ospina-Romero M., Abdiwahab E., Kobayashi L., Filshtein T., Brenowitz W.D., Mayeda E.R., Glymour M.M. (2019). Rate of Memory Change Before and After Cancer Diagnosis. JAMA Netw. Open.

[60] Schilder C.M.T., Seynaeve C., Linn S.C., Boogerd W., Beex L.V.A.M., Gundy C.M., Nortier J.W.R., van de Velde C.J.H., van Dam F.S.A.M., Schagen S.B. (2012). Self-Reported Cognitive Functioning in Postmenopausal Breast Cancer Patients before and during Endocrine Treatment: Findings from the Neuropsychological TEAM Side-Study. Psychooncology.

[61] Freedman R.A., Pitcher B., Keating N.L., Ballman K.V., Mandelblatt J., Kornblith A.B., Kimmick G.G., Hurria A., Winer E.P., Hudis C.A. (2013). Cognitive Function in Older Women with Breast Cancer Treated with Standard Chemotherapy and Capecitabine on Cancer and Leukemia Group B 49907. Breast Cancer Res. Treat..

[62] Keating N.L., Nørredam M., Landrum M.B., Huskamp H.A., Meara E. (2005). Physical and Mental Health Status of Older Long-Term Cancer Survivors. J. Am. Geriatr. Soc..

[63] Mandelblatt J.S., Clapp J.D., Luta G., Faul L.A., Tallarico M.D., McClendon T.D., Whitley J.A., Cai L., Ahles T.A., Stern R.A. (2016). Long-Term Trajectories of Self-Reported Cognitive Function in a Cohort of Older Survivors of Breast Cancer: CALGB 369901 (Alliance). Cancer.

[64] Heflin L.H., Meyerowitz B.E., Hall P., Lichtenstein P., Johansson B., Pedersen N.L., Gatz M. (2005). Cancer as a Risk Factor for Long-Term Cognitive Deficits and Dementia. J. Natl. Cancer Inst..

[65] Hurria A., Goldfarb S., Rosen C., Holland J., Zuckerman E., Lachs M.S., Witmer M., van Gorp W.G., Fornier M., D’Andrea G. (2006). Effect of Adjuvant Breast Cancer Chemotherapy on Cognitive Function from the Older Patient’s Perspective. Breast Cancer Res. Treat..

[66] Stava C., Weiss L.T., Vassilopoulou-Sellin R. (2006). Health Profiles of 814 Very Long-Term Breast Cancer Survivors. Clin. Breast Cancer.

[67] Ibler E., Tran G., Orrell K.A., Serrano L., Majewski S., Sable K.A., Thiede R., Laumann A.E., West D.P., Nardone B. (2018). Inverse Association for Diagnosis of Alzheimer’s Disease Subsequent to Both Melanoma and Non-Melanoma Skin Cancers in a Large, Urban, Single-Centre, Midwestern US Patient Population. J. Eur. Acad. Dermatol. Venereol..

[68] Mahajan A., Chirra M., Dwivedi A.K., Sturchio A., Keeling E.G., Marsili L., Espay A.J. (2020). Skin Cancer May Delay Onset but Not Progression of Parkinson’s Disease: A Nested Case-Control Study. Front. Neurol..

[69] White R.S., Lipton R.B., Hall C.B., Steinerman J.R. (2013). Nonmelanoma Skin Cancer Is Associated with Reduced Alzheimer Disease Risk. Neurology.

[70] Frain L., Swanson D., Cho K., Gagnon D., Lu K.P., Betensky R.A., Driver J. (2017). Association of Cancer and Alzheimer’s Disease Risk in a National Cohort of Veterans. Alzheimers Dement..

[71] Olsen J.H., Friis S., Frederiksen K. (2006). Malignant Melanoma and Other Types of Cancer Preceding Parkinson Disease. Epidemiology.

[72] Driver J.A., Beiser A., Au R., Kreger B.E., Splansky G.L., Kurth T., Kiel D.P., Lu K.P., Seshadri S., Wolf P.A. (2012). Inverse Association between Cancer and Alzheimer’s Disease: Results from the Framingham Heart Study. BMJ.

[73] Klinger D., Hill B.L., Barda N., Halperin E., Gofrit O.N., Greenblatt C.L., Rappoport N., Linial M., Bercovier H. (2021). Bladder Cancer Immunotherapy by BCG Is Associated with a Significantly Reduced Risk of Alzheimer’s Disease and Parkinson’s Disease. Vaccines.

[74] Sun M., Wang Y., Sundquist J., Sundquist K., Ji J. (2020). The Association Between Cancer and Dementia: A National Cohort Study in Sweden. Front. Oncol..

[75] Chen P.C., Muo C.H., Lee Y.T., Yu Y.H., Sung F.C. (2011). Lung Cancer and Incidence of Stroke: A Population-Based Cohort Study. Stroke.

[76] Elbaz A., Peterson B.J., Yang P., van Gerpen J.A., Bower J.H., Maraganore D.M., McDonnell S.K., Ahlskog J.E., Rocca W.A. (2002). Nonfatal Cancer Preceding Parkinson’s Disease: A Case-Control Study. Epidemiology.

[77] Roderburg C., Loosen S.H., Kunstein A., Mohr R., Jördens M.S., Luedde M., Kostev K., Luedde T. (2021). Cancer Patients Have an Increased Incidence of Dementia: A Retrospective Cohort Study of 185,736 Outpatients in Germany. Cancers.

[78] Freedman D.M., Wu J., Chen H., Kuncl R.W., Enewold L.R., Engels E.A., Freedman N.D., Pfeiffer R.M. (2016). Associations between Cancer and Alzheimer’s Disease in a U.S. Medicare Population. Cancer Med..

[79] Realmuto S., Cinturino A., Arnao V., Mazzola M.A., Cupidi C., Aridon P., Ragonese P., Savettieri G., D’Amelio M. (2012). Tumor Diagnosis Preceding Alzheimer’s Disease Onset: Is There a Link between Cancer and Alzheimer’s Disease?. J. Alzheimers Dis..

[80] Aiello Bowles E.J., Walker R.L., Anderson M.L., Dublin S., Crane P.K., Larson E.B. (2017). Risk of Alzheimer’s Disease or Dementia Following a Cancer Diagnosis. PLoS ONE.

[81] Khan N.F., Mant D., Carpenter L., Forman D., Rose P.W. (2011). Long-Term Health Outcomes in a British Cohort of Breast, Colorectal and Prostate Cancer Survivors: A Database Study. Br. J. Cancer.

[82] Hong J.H., Huang C.Y., Chang C.H., Muo C.H., Jaw F.S., Lu Y.C., Chung C.J. (2020). Different Androgen Deprivation Therapies Might Have a Differential Impact on Cognition—An Analysis from a Population-Based Study Using Time-Dependent Exposure Model. Cancer Epidemiol..

[83] Huang W.K., Liu C.H., Pang S.T., Liu J.R., Chang J.W.C., Liaw C.C., Hsu C.L., Lin Y.C., See L.C. (2020). Type of Androgen Deprivation Therapy and Risk of Dementia Among Patients with Prostate Cancer in Taiwan. JAMA Netw. Open.

[84] Jayadevappa R., Chhatre S., Malkowicz S.B., Parikh R.B., Guzzo T., Wein A.J. (2019). Association Between Androgen Deprivation Therapy Use and Diagnosis of Dementia in Men with Prostate Cancer. JAMA Netw. Open.

[85] Jhan J.H., Yang Y.H., Chang Y.H., Guu S.J., Tsai C.C. (2017). Hormone Therapy for Prostate Cancer Increases the Risk of Alzheimer’s Disease: A Nationwide 4-Year Longitudinal Cohort Study. Aging Male.

[86] Krasnova A., Epstein M., Marchese M., Dickerman B.A., Cole A.P., Lipsitz S.R., Nguyen P.L., Kibel A.S., Choueiri T.K., Basaria S. (2020). Risk of Dementia Following Androgen Deprivation Therapy for Treatment of Prostate Cancer. Prostate Cancer Prostatic Dis..

[87] Nead K.T., Gaskin G., Chester C., Swisher-McClure S., Dudley J.T., Leeper N.J., Shah N.H. (2016). Androgen Deprivation Therapy and Future Alzheimer’s Disease Risk. J. Clin. Oncol..

[88] Nead K.T., Gaskin G., Chester C., Swisher-McClure S., Leeper N.J., Shah N.H. (2017). Association Between Androgen Deprivation Therapy and Risk of Dementia. JAMA Oncol..

[89] Shahinian V.B., Kuo Y.-F., Freeman J.L., Goodwin J.S. (2006). Risk of the “Androgen Deprivation Syndrome” in Men Receiving Androgen Deprivation for Prostate Cancer. Arch. Intern. Med..

[90] Tae B.S., Jeon B.J., Shin S.H., Choi H., Bae J.H., Park J.Y. (2019). Correlation of Androgen Deprivation Therapy with Cognitive Dysfunction in Patients with Prostate Cancer: A Nationwide Population-Based Study Using the National Health Insurance Service Database. Cancer Res. Treat..

[91] Baik S.H., Kury F., McDonald C.J. (2017). Risk of Alzheimer’s Disease Among Senior Medicare Beneficiaries Treated With Androgen Deprivation Therapy for Prostate Cancer. J. Clin. Oncol..

[92] Chung S.D., Lin H.C., Tsai M.C., Kao L.T., Huang C.Y., Chen K.C. (2016). Androgen Deprivation Therapy Did Not Increase the Risk of Alzheimer’s and Parkinson’s Disease in Patients with Prostate Cancer. Andrology.

[93] Kao L.T., Lin H.C., Chung S.D., Huang C.Y. (2017). No Increased Risk of Dementia in Patients Receiving Androgen Deprivation Therapy for Prostate Cancer: A 5-Year Follow-up Study. Asian J. Androl..

[94] Khosrow-Khavar F., Rej S., Yin H., Aprikian A., Azoulay L. (2017). Androgen Deprivation Therapy and the Risk of Dementia in Patients with Prostate Cancer. J. Clin. Oncol..

[95] Tae B.S., Jeon B.J., Choi H., Bae J.H., Park J.Y. (2019). Is Androgen Deprivation Therapy Associated with Cerebral Infarction in Patients with Prostate Cancer? A Korean Nationwide Population-Based Propensity Score Matching Study. Cancer Med..

[96] Blanchette P.S., Lam M., Le B., Richard L., Shariff S.Z., Pritchard K.I., Raphael J., Vandenberg T., Fernandes R., Desautels D. (2020). The Association between Endocrine Therapy Use and Dementia among Post-Menopausal Women Treated for Early-Stage Breast Cancer in Ontario, Canada. J. Geriatr. Oncol..

[97] Bromley S.E., Matthews A., Smeeth L., Stanway S., Bhaskaran K. (2019). Risk of Dementia among Postmenopausal Breast Cancer Survivors Treated with Aromatase Inhibitors versus Tamoxifen: A Cohort Study Using Primary Care Data from the UK. J. Cancer Surviv..

[98] Branigan G.L., Soto M., Neumayer L., Rodgers K., Diaz Brinton R. (2020). Association Between Hormone-Modulating Breast Cancer Therapies and Incidence of Neurodegenerative Outcomes for Women with Breast Cancer Key Points. JAMA Netw. Open.

[99] Hong J.C., Kruser T.J., Gondi V., Mohindra P., Cannon D.M., Harari P.M., Bentzen S.M. (2013). Risk of Cerebrovascular Events in Elderly Patients after Radiation Therapy versus Surgery for Early-Stage Glottic Cancer. Int. J. Radiat. Oncol. Biol. Phys..

[100] Smith G.L., Smith B.D., Buchholz T.A., Giordano S.H., Garden A.S., Woodward W.A., Krumholz H.M., Weber R.S., Ang K.K., Rosenthal D.I. (2008). Cerebrovascular Disease Risk in Older Head and Neck Cancer Patients after Radiotherapy. J. Clin. Oncol..

[101] Boulet J., Peña J., Hulten E.A., Neilan T.G., Dragomir A., Freeman C., Lambert C., Hijal T., Nadeau L., Brophy J.M. (2019). Statin Use and Risk of Vascular Events Among Cancer Patients After Radiotherapy to the Thorax, Head, and Neck. J. Am. Heart Assoc..

[102] Du X.L., Cai Y., Symanski E. (2013). Association between Chemotherapy and Cognitive Impairments in a Large Cohort of Patients with Colorectal Cancer. Int. J. Oncol..

[103] Heck J.E., Albert S.M., Franco R., Gorin S.S. (2008). Patterns of Dementia Diagnosis in Surveillance, Epidemiology, and End Results Breast Cancer Survivors Who Use Chemotherapy. J. Am. Geriatr. Soc..

[104] Baxter N.N., Durham S.B., Phillips K.A., Habermann E.B., Virning B.A. (2009). Risk of Dementia in Older Breast Cancer Survivors: A Population-Based Cohort Study of the Association with Adjuvant Chemotherapy. J. Am. Geriatr. Soc..

[105] Du X.L., Xia R., Hardy D. (2010). Relationship between Chemotherapy Use and Cognitive Impairments in Older Women with Breast Cancer: Findings from a Large Population-Based Cohort. Am. J. Clin. Oncol..

[106] Jazzar U., Shan Y., Klaassen Z., Freedland S.J., Kamat A.M., Raji M.A., Masel T., Tyler D.S., Baillargeon J., Kuo Y.F. (2020). Impact of Alzheimer’s Disease and Related Dementia Diagnosis Following Treatment for Bladder Cancer. J. Geriatr. Oncol..

[107] Raji M.A., Tamborello L.P., Kuo Y.F., Ju H., Freeman J.L., Zhang D.D., Giordano S.H., Goodwin J.S. (2009). Risk of Subsequent Dementia Diagnoses Does Not Vary by Types of Adjuvant Chemotherapy in Older Women with Breast Cancer. Med. Oncol..

[108] Fowler M.E., Triebel K.L., Cutter G.R., Schneider L.S., Kennedy R.E. (2020). Progression of Alzheimer’s Disease by Self-Reported Cancer History in the Alzheimer’s Disease Neuroimaging Initiative. J. Alzheimers Dis..

[109] Musicco M., Adorni F., di Santo S., Prinelli F., Pettenati C., Caltagirone C., Palmer K., Russo A. (2013). Inverse Occurrence of Cancer and Alzheimer Disease: A Population-Based Incidence Study. Neurology.

[110] Roe C.M., Fitzpatrick A.L., Xiong C., Sieh W., Kuller L., Miller J.P., Williams M.M., Kopan R., Behrens M.I., Morris J.C. (2010). Cancer Linked to Alzheimer Disease but Not Vascular Dementia. Neurology.

[111] Hanson H.A., Horn K.P., Rasmussen K.M., Hoffman J.M., Smith K.R. (2017). Is Cancer Protective for Subsequent Alzheimer’s Disease Risk? Evidence From the Utah Population Database. J. Gerontol. B Psychol. Sci. Soc. Sci..

[112] Ording A.G., Horváth-Puhó E., Veres K., Glymour M.M., Rørth M., Sørensen H.T., Henderson V.W. (2020). Cancer and Risk of Alzheimer’s Disease: Small Association in a Nationwide Cohort Study. Alzheimers Dement..

[113] Zhu J., Lu D., Sveinsson O., Wirdefeldt K., Fall K., Piehl F., Valdimarsdóttir U., Fang F. (2015). Is a Cancer Diagnosis Associated with Subsequent Risk of Transient Global Amnesia?. PLoS ONE.

[114] Országhová Z., Mego M., Chovanec M. (2021). Long-Term Cognitive Dysfunction in Cancer Survivors. Front. Mol. Biosci..

[115] Joly F., Giffard B., Rigal O., de Ruiter M.B., Small B.J., Dubois M., Lefel J., Schagen S.B., Ahles T.A., Wefel J.S. (2015). Impact of Cancer and Its Treatments on Cognitive Function: Advances in Research from the Paris International Cognition and Cancer Task Force Symposium and Update Since 2012. J. Pain Symptom Manag..

[116] Castel H., Denouel A., Lange M., Tonon M.C., Dubois M., Joly F. (2017). Biomarkers Associated with Cognitive Impairment in Treated Cancer Patients: Potential Predisposition and Risk Factors. Front. Pharmacol..

[117] Handforth C., Clegg A., Young C., Simpkins S., Seymour M.T., Selby P.J., Young J. (2015). The Prevalence and Outcomes of Frailty in Older Cancer Patients: A Systematic Review. Ann. Oncol..

[118] Bluethmann S.M., Mariotto A.B., Rowland J.H. (2016). Anticipating the “Silver Tsunami”: Prevalence Trajectories and Comorbidity Burden among Older Cancer Survivors in the United States. Cancer Epidemiol. Biomark. Prev..

[119] Soto-Perez-De-Celis E., Li D., Yuan Y., Lau M., Hurria A. (2018). Functional versus Chronological Age: Geriatric Assessments to Guide Decision Making in Older Patients with Cancer. Lancet Oncol..

[120] Hurria A., Patel S.K., Mortimer J., Luu T., Somlo G., Katheria V., Ramani R., Hansen K., Feng T., Chuang C. (2014). The Effect of Aromatase Inhibition on the Cognitive Function of Older Patients with Breast Cancer. Clin. Breast Cancer.

[121] Berben L., Floris G., Wildiers H., Hatse S. (2021). Cancer and Aging: Two Tightly Interconnected Biological Processes. Cancers.

[122] Ahles T.A. (2012). Brain Vulnerability to Chemotherapy Toxicities. Psychooncology.

