# Supplementary material for: A Systematic Review on the Potential Acceleration of Neurocognitive Aging in Older Cancer Survivors
Source: Cancers (Basel). 2023 Feb 14;15(4):1215. doi: 10.3390/cancers15041215 (PMC9954467; doi:10.3390/cancers15041215)
Supplement: Supplementary file 1 [file cancers-15-01215-s001.zip › cancers-2153064-supplementary.pdf]

Supplementary Materials

Supplementary S1. Overview of searches in the search engines

1. Search engine: Pubmed

- *Databases*: MEDLINE®, PubMed Central (PMC)
- *Replica of the search*:

  [Neoplasm] *and* TITLE

  [Neurocognition OR neurodegeneration] *and* TITLE/ ABSTRACT

  [Elderly] TITLE/ ABSTRACT

  Limit: “2000 to 2021

  Limit: “languages: English”

  Limit: “Species: Humans”
- *Number of hits from the search*: 2813

2. Search engine: WoS: Web Of Science

- *Databases*: Web of Science Core Collection 1955-present
- *Replica of the search*:

  [Neoplasm] *and* TITLE

  [Neurocognition OR neurodegeneration] *and* TOPIC

  [Elderly] TOPIC

  Limit: “2000-01-01 to 2021-06-12”

  Limit: “document types: Articles”

  Limit: “languages: English”

  Limit: “Not: Web of Science Index: Emerging Sources Citation Index (ESCI) or Book Citation Index - Social Sciences and Humanities (BKCI-SSH) or Book Citation Index - Science (BKCI-S) or Index Chemicus (IC)”
- *Number of hits from the search*: 5925

Supplementary S2. Detailed Search String.

Pubmed

(Neoplasms[Mesh] **OR** neoplas*[ti] **OR** cancer[Mesh] **OR** cancer*[ti] **OR** tumor*[ti] **OR** tumour*[ti] **OR** oncolog*[ti] **OR** “cancer survivors”[Mesh] **OR** glioma*[ti] **OR** Psycho-oncology[Mesh] **OR** psycho-oncolog*[ti] **OR** sarcoma*[ti] **OR** osteosarcoma*[ti] **OR** rhabdomyosarcoma*[ti] **OR** meningeoma*[ti] **OR** leukem*[ti] **OR** AML[ti] **OR** CLL[ti] **OR** CML[ti] **OR** lymphoma*[ti] **OR** melanoma*[ti] **OR** carcinoma*[ti] **OR** germinoma*[ti] **OR** blastoma*[ti] **OR** retinoblastoma*[ti] **OR** neuroblastoma*[ti] **OR** medulloblastoma*[ti] **OR** astrocytoma*[ti] **OR** ependymoma*[ti] **OR** craniopharyngioma*[ti] **OR** chordoma*[ti] **OR** ATRT*[ti] **OR** papilloma*[ti] **OR** neurofibroma*[ti] **OR** non-rhabdomyosarcoma*[ti] **OR** leiomyosarcoma*[ti] **OR** liposarcoma*[ti] **OR** angiosarcoma*[ti] **OR** fibrosarcoma*[ti] **OR** neurofibrosarcoma*[ti] **OR** myosarcoma*[ti] **OR** kaposisarcoma*[ti] **OR** dermatofibrosarcoma*[ti] **OR** chondrosarcoma*[ti] **OR** chemotherap*[ti] **OR** “cranial radiotherap*”[ti] **OR** “cranial irradiation*”[ti] **OR** “cranial radiation*”[ti] **OR** “androgen deprivation*”[ti] **OR** “chemical castration*”[ti] **OR** “androgen block*”[ti] **OR** ADT[ti] **OR** “hormone therap*”[ti] **OR** immunotherap*[ti]) **AND** (“Demyelinating diseases”[Mesh:noexp] **OR** demyelinat*[tiab] **OR** leukoencephalopath*[tiab] **OR** “neurobehavioral manifestations”[Mesh] **OR** “neurological symptom*”[tiab] **OR** neurobehavior*[tiab] **OR** agraphia[tiab] **OR** anomia[tiab] **OR** dyslexi*[tiab] **OR** “speech defic*”[tiab] **OR** “speech disorder*”[tiab] **OR** “speech problem*”[tiab] **OR** “speech disabilit*”[tiab] **OR** “speech dysfunction*”[tiab] **OR** “speech function*”[tiab] **OR** dyscalculi*[tiab] **OR** delirium[tiab] **OR** “mental disabilit*”[tiab] **OR** “mental defic*”[tiab] **OR** “mental retard*”[tiab] **OR** amnesia[Mesh] **OR** amnesia[tiab] **OR** agnosia[tiab] **OR** hallucination*[tiab] **OR** “psychomotor defic*”[tiab] **OR** “psychomotor problem*”[tiab] **OR** “psychomotor disabilit*”[tiab] **OR** “psychomotor dysfunction*”[tiab] **OR** “psychomotor symptom*”[tiab] **OR** “psychomotor function*”[tiab] **OR** apraxia[tiab] **OR** “nervous system disorder*”[tiab] **OR** “brain function*”[tiab] **OR** “brain dysfunction*”[tiab] **OR** “brain disorder*”[tiab] **OR** encephalopath*[tiab] **OR** “auditory defic*”[tiab] **OR** “auditory disorder*”[tiab] **OR** “auditory problem*”[tiab] **OR** “auditory disabilit*”[tiab] **OR** “auditory dysfunction*”[tiab] **OR** “auditory function*”[tiab] **OR** “auditory symptom*”[tiab] **OR** “language defic*”[tiab] **OR** “language disorder*”[tiab] **OR** “language problem*”[tiab] **OR** “language disabilit*”[tiab] **OR** “language dysfunction*”[tiab] **OR** “language symptom*”[tiab] **OR** “language function*”[tiab] **OR** aphasia[tiab] **OR** visuoconstruct*[tiab] **OR** visuopercept*[tiab] **OR** neurodegenerat*[tiab] **OR** neurotoxic*[tiab] **OR** neurocognit*[tiab] **OR** neuropsychology[Mesh] **OR** neuropsych*[tiab] **OR** neuro-psych*[tiab] **OR** cognition[Mesh] **OR** cognition*[tiab] **OR** cognitive*[tiab] **OR** “cognition disorder*”[Mesh] **OR** “cognitive reserve”[Mesh] **OR** “executive function*”[tiab] **OR** neurolinguistic*[tiab] **OR** “problem solving”[tiab] **OR** “spatial navigat*”[tiab] **OR** “neurocognitive disorders”[Mesh] **OR** “mental disorder”[tiab] **OR** “mental proces*”[tiab] **OR** “dissociative disorders”[Mesh] **OR** “dissociative disorder*”[tiab] **OR** mental-deterioration*[tiab] **OR** “mental deterioration*”[tiab] **OR** higher-cerebral-function*[tiab] **OR** “cognitive neuroscience”[Mesh] **OR** CRCI[tiab] **OR** “Chemotherapy-Related Cognitive Impairment”[Mesh] **OR** “cognitive dysfunction”[Mesh] **OR** “Chemo Brain”[Tiab] **OR** Chemobrain[Tiab] **OR** “Chemo fog”[Tiab] **OR** Chemo-fog[Tiab] **OR** “Postoperative Cognitive Complications”[Mesh] **OR** dementia[Mesh] **OR** dement*[tiab] **OR** memory[Mesh] **OR** memory[tiab] **OR** “memory disorders”[Mesh] **OR** attention[Mesh] **OR** “attention defic*”[tiab] **OR** “attention disorder*”[tiab] **OR** “attention problem*”[tiab] **OR** “attentional

disabilit*”[tiab] **OR** “attention dysfunction*”[tiab] **OR** “attention symptom*”[tiab] **OR** “attention function*”[tiab] **OR** “executive defic*”[tiab] **OR** “executive disorder*”[tiab] **OR** “executive problem*”[tiab] **OR** “executive disabilit*”[tiab] **OR** “executive dysfunction*”[tiab] **OR** “executive symptom*”[tiab] **OR** “executive function*”[tiab] **OR** “executive function”[Mesh] **OR** “verbal fluenc*”[tiab] **OR** “processing speed*”[tiab] **OR** “brain damage*”[tiab] **OR** “brain injur*”[tiab] **OR** “neurofibrillary tangle*”[tiab] **OR** tauopathies[Mesh] **OR** tauopath*[tiab] **OR** “amyloid*”[tiab] **OR** “neurofilament*”[tiab] **OR** “cerebrovascular disorder*”[tiab] **OR** “cerebrovascular disease*”[tiab] **OR** “brain ischemia*”[tiab] **OR** “cerebral ischemia*”[tiab] **OR** “brain hypoxia*”[tiab] **OR** “cerebral hypoxia*”[tiab] **OR** “brain infarction*”[tiab] **OR** “ischemic attack”[tiab] **OR** “intracranial embolism*”[tiab] **OR** “intracranial thrombosis”[tiab] **OR** leukomalacia[tiab]) **AND** (elderly[tiab] **OR** ageing[tiab] **OR** aging[tiab] **OR** postmenopausal[tiab] **OR** post-menopausal[tiab] **OR** "older age*"[tiab] **OR** "old age*"[tiab] **OR** "older adult*"[tiab] **OR** "older patient*"[tiab] **OR** "older survivor*"[tiab] **OR** "older participant*"[tiab] **OR** "older population*"[tiab] **OR** "older men"[tiab] **OR** "older women"[tiab] **OR** "older cohort*"[tiab])

<u>Web of Science (WoS)</u>

TI=(Neoplas* **OR** cancer* **OR** tumor* **OR** tumour* **OR** oncolog* **OR** glioma* **OR** psycho-oncolog* **OR** sarcoma* **OR** osteosarcoma* **OR** rhabdomyosarcoma* **OR** meningeoma* **OR** leukem* **OR** AML **OR** CLL **OR** CML **OR** lymphoma* **OR** melanoma* **OR** carcinoma* **OR** germinoma* **OR** blastoma* **OR** retinoblastoma* **OR** neuroblastoma* **OR** medulloblastoma* **OR** astrocytoma* **OR** ependymoma* **OR** craniopharyngioma* **OR** chordoma* **OR** ATRT* **OR** papilloma* **OR** neurofibroma* **OR** non-rhabdomyosarcoma* **OR** leiomyosarcoma* **OR** liposarcoma* **OR** angiosarcoma* **OR** fibrosarcoma* **OR** neurofibrosarcoma* **OR** myosarcoma* **OR** kaposisarcoma* **OR** dermatofibrosarcoma* **OR** chondrosarcoma* **OR** chemotherap* **OR** “cranial radiotherap*” **OR** “cranial irradiation*” **OR** “cranial radiation*” **OR** “androgen deprivation*” **OR** “chemical castration*” **OR** “androgen block*” **OR** ADT **OR** “hormone therap*” **OR** immunotherap*) AND TS=(demyelinat* **OR** leukoencephalopath* **OR** “neurological symptom*” **OR** neurobehavior* **OR** agraphia **OR** anomia **OR** dyslexi* **OR** “speech defic*” **OR** “speech disorder*” **OR** “speech problem*” **OR** “speech disabilit*” **OR** “speech dysfunction*” **OR** “speech function*” **OR** dyscalculi* **OR** delirium **OR** “mental disabilit*” **OR** “mental defic*” **OR** “mental retard*” **OR** amnesia **OR** agnosia **OR** hallucination* **OR** “psychomotor defic*” **OR** “psychomotor problem*” **OR** “psychomotor disabilit*” **OR** “psychomotor dysfunction*” **OR** “psychomotor symptom*” **OR** “psychomotor function*” **OR** apraxia **OR** “nervous system disorder*” **OR** “brain function*” **OR** “brain dysfunction*”[tiab] **OR** “brain disorder*” **OR** encephalopath* **OR** “auditory defic*” **OR** “auditory disorder*” **OR** “auditory problem*” **OR** “auditory disabilit*” **OR** “auditory dysfunction*” **OR** “auditory function*” **OR** “auditory symptom*” **OR** “language defic*” **OR** “language disorder*” **OR** “language problem*” **OR** “language disabilit*” **OR** “language dysfunction*” **OR** “language symptom*” **OR** “language function*” **OR** aphasia **OR** visuoconstruct* **OR** visuopercept* **OR** neurodegenerat* **OR** neurotoxic* **OR** neurocognit* **OR** neuropsych* **OR** neuro-psych* **OR** cognition* **OR** cognitive* **OR** “executive function*” **OR** neurolinguistic* **OR** “problem solving” **OR** “spatial navigat*” **OR** “mental disorder” **OR** “mental proces*” **OR** “dissociative disorder*” **OR** mental-deterioration* **OR** “mental deterioration*” **OR** higher-cerebral-function* **OR** CRCI **OR** “Chemo Brain” **OR** Chemobrain **OR** “Chemo fog” **OR** Chemo-fog **OR** dement* **OR** memory **OR** “attention defic*” **OR** “attention disorder*” **OR** “attention problem*” **OR** “attentional disabilit*” **OR** “attention dysfunction*” **OR** “attention symptom*” **OR** “attention function*” **OR** “executive defic*” **OR** “executive disorder*” **OR** “executive problem*” **OR** “executive disabilit*” **OR** “executive dysfunction*” **OR** “executive symptom*” **OR** “executive function*” **OR**

“verbal fluenc*” **OR** “processing speed*” **OR** “brain damage*” **OR** “brain injur*” **OR** “neurofibrillary tangle*” **OR** tauopath* **OR** “amyloid*” **OR** “neurofilament*” **OR** “cerebrovascular disorder*” **OR** “cerebrovascular disease*” **OR** “brain ischemia*” **OR** “cerebral ischemia*” **OR** “brain hypoxia*” **OR** “cerebral hypoxia*” **OR** “brain infarction*” **OR** “ischemic attack” **OR** “intracranial embolism*” **OR** “intracranial thrombosis” **OR** leukomalacia) **AND** TS=(elderly **OR** ageing **OR** aging **OR** postmenopausal **OR** post-menopausal **OR** "older age*" **OR** "old age*" **OR** "older adult*" **OR** "older patient*" **OR** "older survivor*" **OR** "older participant*" **OR** "older population*" **OR** "older men*" **OR** "older women*" **OR** "older cohort*")
